# A case for improved assessment of gut permeability: a meta-analysis quantifying the lactulose:mannitol ratio in coeliac and Crohn’s disease

**DOI:** 10.1186/s12876-021-02082-z

**Published:** 2022-01-10

**Authors:** Jonathan Gan, Scarlet Nazarian, Julian Teare, Ara Darzi, Hutan Ashrafian, Alex J. Thompson

**Affiliations:** 1 Department of Surgery and Cancer, Institute of Global Health Innovation, St Mary’s Hospital, Imperial College London, 10th Floor, Queen Elizabeth the Queen Mother WingSouth Wharf Road, London, W2 1NY UK; 2 Hamlyn Centre for Robotic Surgery, St Mary’s Hospital, Imperial College London, Level 3 Paterson BuildingSouth Wharf Road, London, W2 1NY UK

**Keywords:** Coeliac, Crohn’s disease, Lactulose Mannitol test, Gut permeability

## Abstract

**Background:**

A widely used method in assessing small bowel permeability is the lactulose:mannitol test, where the lactulose:mannitol ratio (LMR) is measured. However, there is discrepancy in how the test is conducted and in the values of LMR obtained across studies. This meta-analysis aims to determine LMR in healthy subjects, coeliac and Crohn’s disease.

**Methods:**

A literature search was performed using PRISMA guidance to identify studies assessing LMR in coeliac or Crohn’s disease. 19 studies included in the meta-analysis measured gut permeability in coeliac disease, 17 studies in Crohn’s disease. Outcomes of interest were LMR values and comparisons of standard mean difference (SMD) and weighted mean difference (WMD) in healthy controls, inactive Crohn’s, active Crohn’s, treated coeliac and untreated coeliac. Pooled estimates of differences in LMR were calculated using the random effects model.

**Results:**

Pooled LMR in healthy controls was 0.014 (95% CI: 0.006–0.022) while pooled LMRs in untreated and treated coeliac were 0.133 (95% CI: 0.089–0.178) and 0.037 (95% CI: 0.019–0.055). In active and inactive Crohn’s disease, pooled LMRs were 0.093 (95% CI: 0.031–0.156) and 0.028 (95% CI: 0.015–0.041). Significant differences were observed in LMR between: (1) healthy controls and treated coeliacs (SMD = 0.409 95% CI 0.034 to 0.783, *p* = 0.032), (2) healthy controls and untreated coeliacs (SMD = 1.362 95% CI: 0.740 to 1.984, *p* < 0.001), (3) treated coeliacs and untreated coeliacs (SMD = 0.722 95% CI: 0.286 to 1.157, *p* = 0.001), (4) healthy controls and inactive Crohn’s (SMD = 1.265 95% CI: 0.845 to 1.686, *p* < 0.001), (5) healthy controls and active Crohn’s (SMD = 2.868 95% CI: 2.112 to 3.623, *p* < 0.001), and (6) active Crohn’s and inactive Crohn’s (SMD = 1.429 (95% CI: 0.580 to 2.278, *p* = 0.001). High heterogeneity was observed, which was attributed to variability in protocols used across different studies.

**Conclusion:**

The use of gut permeability measurements in screening and monitoring of coeliac and Crohn’s disease is promising. LMR is useful in performing this function with significant limitations. More robust alternative tests with higher degrees of clinical evidence are needed if measurements of gut permeability are to find widespread clinical use.

**Supplementary Information:**

The online version contains supplementary material available at 10.1186/s12876-021-02082-z.

## Background

There is emerging evidence that disturbances in gut barrier function play an important role in gastrointestinal (GI) diseases such as coeliac disease, inflammatory bowel disease (IBD), environmental enteric dysfunction, and in conditions outside the GI tract such as schizophrenia, autism and Parkinsons Disease [1, 2]. Current established methods of measuring gut permeability in patients include the lactulose:mannitol (L:M) test, lactulose:rhamnose (L:R) test, chromium-51 labelled ethylenediamine tetraacetic acid (Cr-EDTA) assay, polyethylene glycol (PEG) test, use of Ussing chambers, analysis of haematological markers such as zonulin, and analysis of bacterial markers such as systemic lipopolysaccharide (LPS) [2]. Despite the various options available to measure gut permeability, their use in clinical settings is still limited [2].

For the practising clinician, a reliable gut permeability assay could potentially provide a new way to monitor established diseases such as IBD and coeliac disease, and to develop a better understanding of functional gut disorders (FGDs). FGD is currently used as a ‘catch-all’ term for poorly understood gastrointestinal conditions and treated as a diagnosis of exclusion. The impact of FGD on health systems is not to be underestimated. Globally, it affects 11% of the population and accounts for 20% to 50% of gastroenterology outpatient work [3]. A better appreciation of the link between gut permeability and FGD would aid the clinician in tackling this multi-faceted condition in a more effective manner.

One of the widely used methods to measure small bowel permeability is the L:M test, in which, after a period of fasting, subjects are asked to drink a solute containing the two sugars lactulose and mannitol. Urinary excretion of both lactulose and mannitol are then measured several hours after ingestion of solute, and the lactulose:mannitol ratio (LMR) is calculated as an indicator of permeability [4].

The L:M test is useful as both sugars are passively absorbed from the intestine, not extensively metabolised, and excreted unchanged in urine in proportion to the quantities absorbed [4]. The smaller sugar alcohol molecule (mannitol) is assumed to permeate transcellularly through the water pores of the membrane, whereas the larger disaccharide molecule (lactulose) is assumed to permeate paracellularly through the tight junctions [5]. In states of increased gut permeability, lactulose would traverse through the paracellular spaces, cleared by glomerular filtration, not undergo selective reabsorption, and present itself in higher levels in urine, thus leading to an increased LMR.

The L:M test is thought to be a good representative of gut permeability as measurements using a single molecule do not account for confounding factors such as intestinal transit time, gastric emptying rate, renal/hepatic function or total urinary excretion [6]. By taking the ratio of excretion of two molecules, the effects of these confounding factors can be eliminated [7].

Although the L:M test has been in use since the 1970s [8], it suffers from limitations, particularly in subjects where longitudinal urine collection is challenging (e.g. infants [9] and patients with reduced urinary output [10]). Furthermore, absolute LMR values for the small bowel in healthy subjects and in disease are not yet established. To address this issue, we performed a meta-analysis to quantify LMR values in healthy participants and in various states of coeliac and Crohn’s disease, two conditions in which altered gut permeability is observed. The results of this meta-analysis are presented below, and variations in the methods used to conduct the L:M test are also explored. To the best of our knowledge, there are not many meta-analyses presented on the L:M test in coeliac and Crohn’s disease. The results highlight the limitations of the test and the improvements required to bring measurements of gut permeability into larger scale clinical use.

## Methods

A systematic review was conducted according to the recommendations in the Preferred Reporting Items for Systematic Reviews and Meta-Analyses (PRISMA) statement [11]. This study has been registered with the International Prospective Register of Systematic Reviews (PROSPERO) (PROSPERO ID CRD42021259836). Prior to conducting the meta-analysis, the eligibility criteria, description of intervention, and comparison and outcome of interest were established. The literature search was conducted by two independent reviewers.

### Eligibility criteria

We included all observational studies, cross-sectional studies, cohort studies and trials pertaining coeliac and Crohn’s disease. We excluded papers that did not report absolute LMR values as well as in vitro studies, animal studies, and studies where sample groups were mixed (e.g. Crohn’s plus ulcerative colitis). Studies had to be published in peer reviewed journals and the search was not restricted by language.

### Literature identification

In January 2020, with the help of a medical librarian, literature searches were conducted using the following databases: Embase (1988–2020); Ovid MEDLINE In Process & Other Non-Indexed Citations and Ovid MEDLINE (1946 to Present); and Cochrane Database of Systematic Reviews. Terms and/or abbreviations to describe coeliac, Crohn’s disease and gut permeability were combined into a search strategy textbox. Free-text terms were then used in various combinations to ensure a complete search in the databases mentioned above. The combined searches relating to gut permeability in coeliac and Crohn’s disease were explored using the terms ‘and’ and 'or’. The following search terms were used: ‘‘permeability’’, ‘‘leaky or leakiness’’, ‘‘lactulose mannitol’’, ‘‘inflammatory bowel disease or IBD’’, ‘Crohn’s Disease’, ‘coeliac disease’, ‘Crohn’s’’, and ‘‘coeliac’’. The search strategies are presented in Additional file 1: Appendix 1, 2 and 3.

### Study selection

The titles and abstracts of the search were then screened. Full text articles or abstracts of potentially relevant references in the articles also underwent review. Conference abstracts and papers with no full texts available were excluded. The study selection process was performed by two independent reviewers who were blinded (JG and SN), and any disagreements were resolved by a third author (HA). JG and SN appraised quality independently.

### Data extraction

The following information was extracted from the studies chosen by JG and SN: number of patients, study objectives, study methodology, results, type of population, L:M study protocol, type of solute given to subjects, urine collection time, method of urine analysis and LMR values. Data extracted were then tabulated in Microsoft Excel and the study outcomes were reviewed by a third reviewer (AT).

The primary study outcomes were Standardised Mean Differences (SMD) of LMR values in healthy controls, treated coeliac patients, untreated coeliac patients, patients with active Crohn’s disease, and patients with inactive Crohn’s disease. The secondary outcomes were comparisons of use of different concentrations of lactulose and mannitol (5 parts lactulose to 2 parts mannitol, and 2 parts lactulose to 1 part mannitol) in the aforementioned groups.

### Risk of bias assessment

Randomised control trials (RCTs) were assessed using the Cochrane Risk of Bias tool [12], while non-randomised trials were assessed using the Risk Of Bias In Non-randomised Studies of Interventions tool (ROBINS-I) [13]. ROBINS-I was also used for cohort studies as per Cochrane recommendations [14]. The Newcastle Ottawa Score (NOS) was used for case control and cross sectional studies [15]. The risk of bias in all studies was assessed by two independent reviewers. The parameters assessed in cohort and case control studies included study design, outcome measurements, representativeness of cases, control selections, ascertainment of exposure, and follow up rate. Using the ROBINS-I assessment tool, potential bias was assessed pre-intervention, at intervention and post intervention. For studies assessed using the NOS tool, a single bias value was calculated for each study.

In the NOS assessment, a score of ≤ 3 indicates poor quality, 4–6 moderate quality, and ≥ 7 good quality. The ROBINS-I tool ranks the risk of bias in studies as ‘low risk’, ‘moderate risk’, ‘serious risk’, ‘critical risk’ or ‘no information’. The findings from the two assessors were evaluated by a third reviewer.

All three modes of assessment were performed by J.G and S.N, and any disagreements were resolved by consensus and discussion with the other authors.

### Statistical analysis

Stata 15 (StataCorp. 2017. Stata Statistical Software: Release 15. College Station, TX: StataCorp LLC) was used for the statistical analysis reported in this study. All analyses were performed using a random effects model in response to expected heterogeneity of data collected. In order to calculate weighted mean and standard mean values of LMR, values of LMR reported in individual studies were pooled into healthy controls, patients with untreated coeliac disease, patients with treated coeliac disease, patients with active Crohn’s and patients with inactive Crohn’s disease. The definition of active Crohn’s and inactive Crohn’s in individual papers are listed in Additional file 1: Table S1.

The Weighted Mean Difference (WMD) and Standard Mean Difference (SMD) of LMR were calculated between controls and treated coeliac, controls and untreated coeliac, treated and untreated coeliac, controls and inactive Crohn’s disease, controls and active Crohn’s disease, and active and inactive Crohn’s disease. This approach calculated overall weighted mean and standard mean differences (i.e. incorporating all relevant studies included in this meta-analysis) and also ascribed an individual weighted mean and standard mean difference for each individual study with 95% Confidence Intervals (CIs). The weighted mean differences refer to the pooled estimates, with the 'weighted' component referring to the different weights applied to each study (or each patient/participant group) in the overall calculation. This was done to accommodate the different sizes (participant number) of studies included in this meta-analysis and the different sizes of patient/participant groups within studies. Weighted mean differences and weighted mean values were calculated in line with principles set out by Egger et al. [16].

Meta-analysis of summary estimates of proportions was also calculated for overall LMRs and sensitivities and specificities using conventional methodology. All results for pooled estimates were presented with 95% CIs. I^2^ values were calculated to assess heterogeneity. *P* values were calculated using the chi-squared test, and the results were considered significant for *P* < 0.05. We note that for studies in which paired data were reported (i.e. where LM ratios were measured before and after treatment), pre- and post-treatment LMR values were simply allocated into the appropriate groups (i.e. paired data was not treated differently to unpaired data).

A bivariate model for diagnostic meta-analysis was used to compute pooled sensitivity and specificity data where available. The relationship between sensitivity and specificity was assessed using a hierarchical summary receiver operating characteristic (SROC) model. SROC curves were utilized to convey the diagnostic test performance and a prediction region curve was also plotted. Trapezoidal integration was used to calculate the pooled area under the curve (AUC), where 0.5 implies that a test was equally likely to diagnose a positive result as either positive or negative and a value of 1.0 indicates a ‘perfect’ test that gives a 100% correct diagnosis. A pooled AUC value of 0.75 or above represents a test with good accuracy [17]. The ‘gold standard’ test that we used for the sensitivity and specificity assessment was intestinal biopsy. It is noted that this dataset is the only aspect of the study analysed using Diagnostic Test Accuracy (DTA) methodology and hence the results in this paper as a whole was analysed as a conventional meta-analysis.

## Results

### Search results and study description

After performing general searches for the relevant subjects and combining them, 377 abstracts were extracted from Medline, 569 abstracts were found in Embase and 190 in the Cochrane. After duplicates were removed, we found that the total number of relevant abstracts was 633.

50 abstracts were found to be relevant to coeliac disease. Of these, a further 31 studies were excluded as per the exclusion criteria discussed above, meaning that a total of 19 coeliac disease studies were selected for analysis. We found that 97 studies were relevant to Crohn’s disease. Of these, 80 studies were excluded according to the exclusion criteria discussed above. Hence, a total of 17 Crohn’s disease studies were included in this meta-analysis. These results are summarised in the PRISMA flow diagrams (Additional file 1: Fig S1 and S2).

The selected studies pertaining gut permeability in Crohn’s disease and coeliac disease are tabulated in Additional file 1: Tables S1 and S2 respectively [18–51]. Altogether, there were 15 studies that investigated gut permeability in Crohn's disease specifically using the L:M test, 17 studies that investigated gut permeability in coeliac disease specifically using the L:M test, and 2 studies that investigated both Crohn’s and coeliac using the L:M test.

### Meta-analysis of results

For the studies included in this meta-analysis, the average mean ages (i.e. the average value of the mean ages reported in each study) and average age ranges (i.e. average minimum age to average maximum age) reported for healthy controls, patients with Crohn’s disease, and patients with coeliac disease were 28.02 (range: 15.2–44.8) years, 30.5 (range: 16.2–49.2) years and 32.6 (range: 14.7–50.7) years respectively. The median LMR values and weighted and standard mean difference values calculated for each study are presented in Tables 1 and 2. In studies where the median LMR value was unavailable, the mean value was used. Standard deviation values associated with each LMR presented in Tables 1 and 2 were either as stated in the individual studies, or if unavailable, were derived from the published range, interquartile range (IQR), 95% CI or standard error of mean (SEM) using established statistical methods [52, 53].

**Table 1:** Summary of number (no) of patients/participants and lactulose:mannitol ratios (LMRs) reported in untreated coeliac patients, treated coeliac patients, and healthy controls

Study	Outcome	No of healthy controls	Reported LMR in healthy controls	Standard Deviation	No of untreated coeliac patients	Reported LMR in untreated coeliac	Standard Deviation	No of treated coeliac patients	Reported LMR in treated coeliac patients	Standard Deviation	Statistically calculated WMD (95% CI)	Statistically calculated SMD (95% CI)
Elia et al. 1991	Control versus untreated coeliac	24	0.021*	0.020	15	0.152*	0.120				0.131 (0.070, 0.192)	1.736 (0.979, 2.492)
Vogelsang et al. 2001	Treated versus untreated coeliac				16	0.12	0.058	19	0.028	0.028	0.092 (0.061, 0.123)	2.085 (1.252, 2.919)
Kuitunen et al. 1996	Control versus untreated coeliac	18	0.030	0.043	22	0.260	0.410				0.230 (0.058, 0.402)	0.751 (0.106, 1.397)
Kuitunen et al. 1996	Control versus treated coeliac	18	0.030	0.043				17	0.040	0.010	0.010 (-0.010, 0.030)	0.316 (-0.351, 0.983)
Kuitunen et al. 1996	Treated versus untreated coeliac				22	0.260	0.410	17	0.040	0.010	0.220 (0.049, 0.391)	0.712 (0.059, 1.365)
Ukabam et al. 1985	Control versus untreated coeliac	25	0.009	0.003	13	0.110	0.156				0.101 (0.016, 0.186)	1.121 (0.402, 1.839)
Ukabam et al. 1985	Control versus treated coeliac	25	0.009	0.003				13	0.016	0.016	0.007 (-0.002, 0.016)	0.728 (0.037, 1.419)
Ukabam et al. 1985	Treated versus untreated coeliac				13	0.110	0.156	13	0.016	0.016	0.094 (0.009, 0.179)	0.848 (0.042, 1.653)
Vilela et al. 2008	Control versus treated coeliac	15	0.003	0.003				22	0.013	0.016	0.010 (0.003, 0.017)	0.785 (0.103, 1.466)
Hamilton et al. 1987	Control versus untreated coeliac	33	0.036	0.021	4	0.296*	1.308				0.264 (-1.018, 1.546)	0.688 (-0.362, 1.739)
Marsilio et al. 1998	Control versus untreated coeliac	30	0.024*	0.006	10	0.072*	0.025				0.048 (0.032, 0.064)	3.623 (2.539, 4.708)
Van Elburg et al. 1993	Control versus untreated coeliac	22	0.043*	0.030	9	0.243*	0.100				0.200 (0.133, 0.267)	3.425 (2.251, 4.599)
Rajani et al. 2016	Control versus untreated coeliac	26	0.022	0.016	65	0.043	0.070				0.021 (0.003, 0.039)	0.351 (-0.106, 0.809)
Rajani et al. 2016	Control versus treated coeliac	26	0.022	0.016				47	0.024	0.077	0.002 (-0.021, 0.025)	0.032 (-0.447, 0.511)
Rajani et al. 2016	Treated versus untreated coeliac				65	0.043	0.070	47	0.024	0.077	0.019 (0.009, 0.047)	0.261 (0.155–0.638)
Smecuol et al. 1997	Treated versus untreated coeliac				27	0.360*	0.380	15	0.130*	0.188	0.230 (0.058, 0.402)	0.706 (0.056, 1.356)
Smecuol et al. 2005	Control versus untreated coeliac	30	0.017*	0.040	30	0.073*	0.090				0.056 (0.021, 0.091)	0.804 (0.277, 1.331)
Juby et al. 1989	Control versus untreated coeliac	12	0.016*	0.007	17	0.163*	0.313				0.147 (-0.002, 0.296)	0.610 (-0.147, 1.367)
Novacek et al. 1999a	Untreated coeliac disease with normal liver function tests				106	0.110	0.315					
Novacek et al. 1999b	Untreated coeliac disease with abnormal liver function tests before versus after gluten free diet				72	0.340	1.400	64	0.050	0.070	0.290 (0.034, 0.614)	0.284 (0.054, 0.623)
Vecsei et al. 2009	Treated versus untreated coeliac				47	0.177		47	0.053			
Johnston et al. 2000	Control versus untreated coeliac	21	0.013*		16	0.105*						
Johnston et al. 2000	Control versus treated coeliac	21	0.013*					7	0.013*			
Johnston et al. 2000	Treated versus untreated coeliac				16	0.105*		7	0.013*			
Catassi et al. 1997	Control versus untreated coeliac	54	0.014		29	0.038						
Smecuol et al. 1999	Untreated coeliac disease				12	0.101	0.069					
Smecuol et al. 2013a	Untreated coeliac disease				10	0.110	0.159					
Smecuol et al. 2013b	Untreated coeliac disease				12	0.054	0.440					
Gatti et al. 2013a	Treated coeliac disease							75	0.055	0.04		
Gatti et al. 2013b	Treated coeliac disease							96	0.052	0.055		

Where appropriate data was available, the calculated weighted mean difference (WMD) and standard mean difference (SMD) in LMR (between the coeliac cohort and healthy controls) are also shown. * = mean value, ** = unknown if value is mean or median, = Standard deviation (SD) calculated from range, = SD calculated from interquartile range (IQR), = SD calculated from standard error of mean (SEM), = SD calculated from 95% confidence interval (CI)

**Table 2:** Summary of number (no) of patients/participants and lactulose:mannitol ratios (LMRs) reported in patients with active Crohn’s disease, patients with inactive Crohn’s disease, and healthy controls

Study	Outcome	Number of healthy controls	Reported LMR in healthy controls	Standard deviation	Number of active Crohn’s patients	Reported LMR in active Crohn's	Standard deviation	Number of inactive Crohn’s patients	Reported LMR in inactive Crohn's	Standard deviation	Statistically calculated WMD (95% CI)	Statistically calculated SMD (95% CI)
Marsilio et al. 1998	Control versus active Crohn’s	30	0.024*	0.006	10	0.200*	0.082				0.176 (0.125, 0.227)	4.373 (3.157, 5.589)
Vilela et al. 2008	Control versus inactive Crohn’s	15	0.003	0.003				31	0.021	0.006	0.018 (0.015, 0.021)	3.467 (2.516, 4.418)
Dastych et al. 2008	Control versus active Crohn’s	20	0.012*	0.008	20	0.076*	0.037				0.064 (0.047, 0.081)	2.396 (1.575, 3.217)
D'Inca et al. 2006	Control versus inactive Crohn’s versus first degree relatives	35	0.01	0.003				115	0.03	0.045	0.020 (0.012, 0.028)	0.506 (0.123, 0.889)
Wild et al. 2003	Control versus inactive Crohn’s patients after 10 weeks of tapering steroids who eventually relapsed	23	0.021*	0.004				11	0.055*	0.018	0.05 (0.04, 0.07)	3.209 (2.144, 4.274)
Wild et al. 2003	Control versus inactive Crohn’s patients after 10 weeks of tapering steroids who eventually did not relapse	23	0.021*	0.004				11	0.026*	0.017		0.497 (-0.232, 1.225)
Wild et al. 2003	Control versus active Crohn’s patients	23	0.021*	0.004	30	0.088*	0.026				0.067 (0.058, 0.076)	3.387 (2.534, 4.240)
Wild et al. 2003	Active Crohn’s patients versus inactive Crohn’s patients after 10 weeks of tapering steroids who eventually relapsed				30	0.088*	0.026	11	0.055*	0.018	0.033 (0.019, 0.047)	1.364 (0.609, 2.118)
Wild et al. 2003	Active Crohn’s patients versus inactive Crohn’s patients after 10 weeks of tapering steroids who eventually did not relapse				30	0.088*	0.026	11	0.026*	0.017	0.062 (0.048, 0.076)	2.582 (1.684, 3.479)
Garcia Vilela et al. 2008	Control versus inactive Crohn’s	15	0.005*	0.004				31	0.021*	0.010	0.016 (0.012, 0.020)	1.879 (1.148, 2.609)
Sigalet et al. 2013	Control versus active Crohn’s	10	0.029*	0.008	7	0.056*	0.025				0.026 (0.007, 0.045)	1.531 (0.421, 2.641)
Sigalet et al. 2013	Control versus inactive Crohn’s	10	0.029*	0.008				7	0.032*	0.010	0.002 (-0.007, 0.011)	0.226 (-0.743, 1.195)
Sigalet et al. 2013	Active versus inactive Crohn’s				7	0.056*	0.025	7	0.032*	0.010	0.024 (0.004, 0.044)	1.261 (0.098, 2.423)
Zamora et al. 1999	Control versus inactive Crohn’s	21	0.019	0.01				14	0.027	0.040	0.008 (-0.013,0.030)	0.313 (-0.368, 0.993)
Andre et al. 1988	Control versus active Crohn’s	100	0.021*	0.01	15	0.132*	0.11				0.111 (0.055, 0.167)	2.787 (2.134, 3.440)
Andre et al. 1988	Control versus inactive Crohn’s	100	0.021*	0.01				15	0.074*	0.090	0.053 (0.007,0.099)	1.604 (1.023, 2.186)
Andre et al. 1988	Active versus inactive Crohn’s				15	0.132*	0.11	15	0.074*	0.090	0.058 (-0.014, 0.130)	0.577 (-0.154, 1.309)
Sturniolo et al. 2001	Inactive Crohn’s							12	0.041*	0.010		
Buhner et al. 2006	Control versus inactive Crohn’s versus first degree relatives versus non-blood relatives	96	0.015	0.005				128	0.026	0.016	0.011 (0.008, 0.014)	0.877 (0.601, 1.154)
D'Inca et al. 1999	Control versus Inactive Crohn’s patients who eventually relapsed	80	0.009*	0.004				52	0.045*	0.042	0.036 (0.025, 0.047)	1.359 (0.973, 1.745)
D'Inca et al. 1999	Control versus Inactive Crohn’s patients who eventually did not relapse	80	0.009*	0.004				78	0.027*	0.027	0.018 (0.012, 0.024)	0.938 (0.610, 1.267)
Swanson et al. 2011	Control versus inactive Crohn’s	7	0.094^**^					6	0.085^**^			
Benjamin et al. 2012a	Inactive Crohn’s							15	0.067	0.024		
Benjamin et al. 2012b	Inactive Crohn’s							15	0.071	0.061		
Hilsden et al. 1996	Controls versus first degree relatives of Crohn’s patients in remission	26	0.017*	0.006								
Hilsden et al. 1999	Inactive Crohn’s							61	0.018*	0.011		
Breslin et al. 2001	Controls versus spouses of patients with inactive Crohn’s	26	0.017*	0.005								

Where appropriate data was available, the calculated weighted mean difference (WMD) and standard mean difference (SMD) in LMR (between the Crohn’s cohort and healthy controls) are also shown. * = mean value, ** = unknown if value is mean or median, = Standard deviation (SD) calculated from range, = SD calculated from interquartile range (IQR), = SD calculated from standard error of mean (SEM), = SD calculated from 95% confidence interval (CI)

**Fig. 1:**
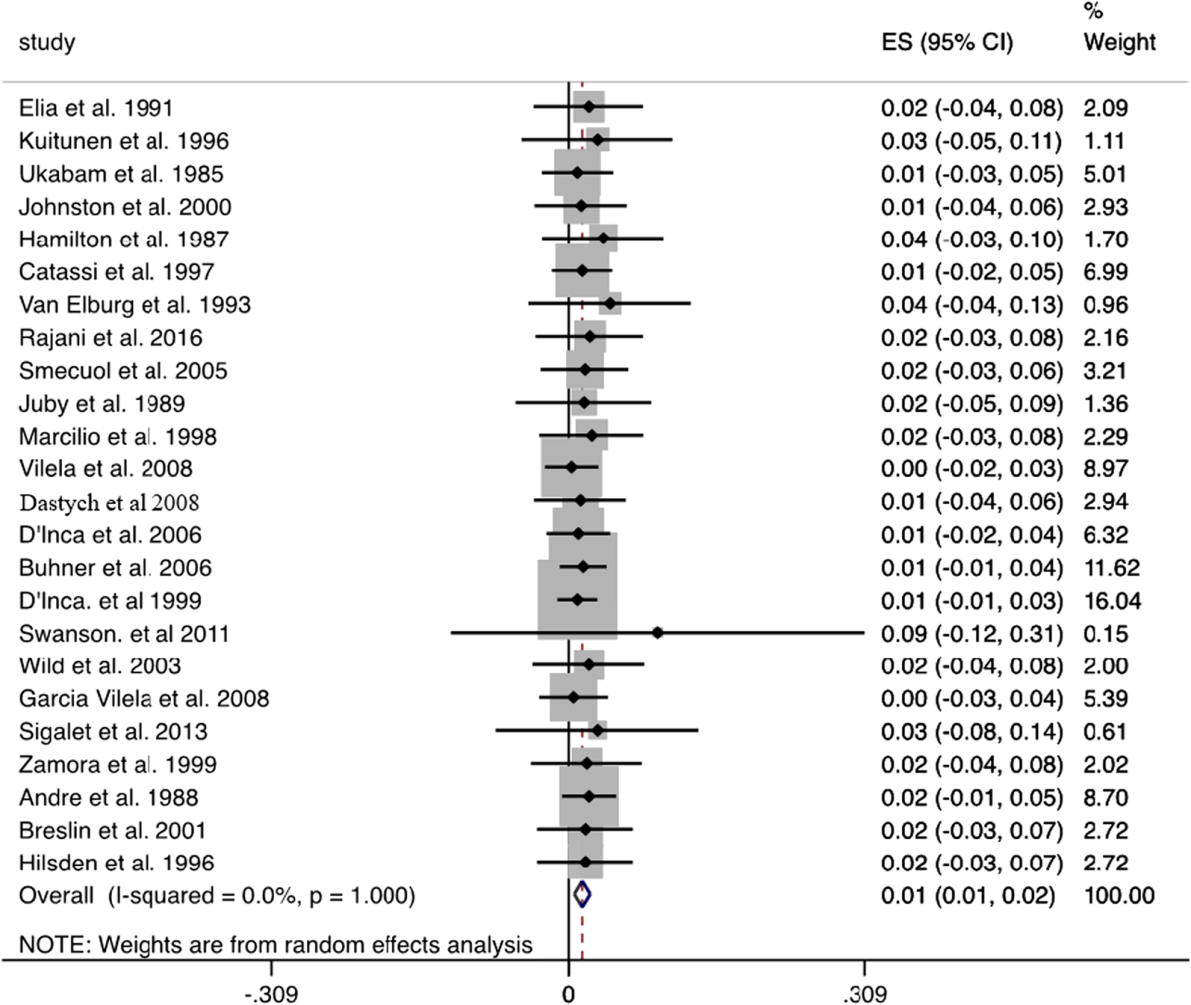
Forest plot showing pooled LMR values (weighted mean) in healthy subjects

**Fig. 2:**
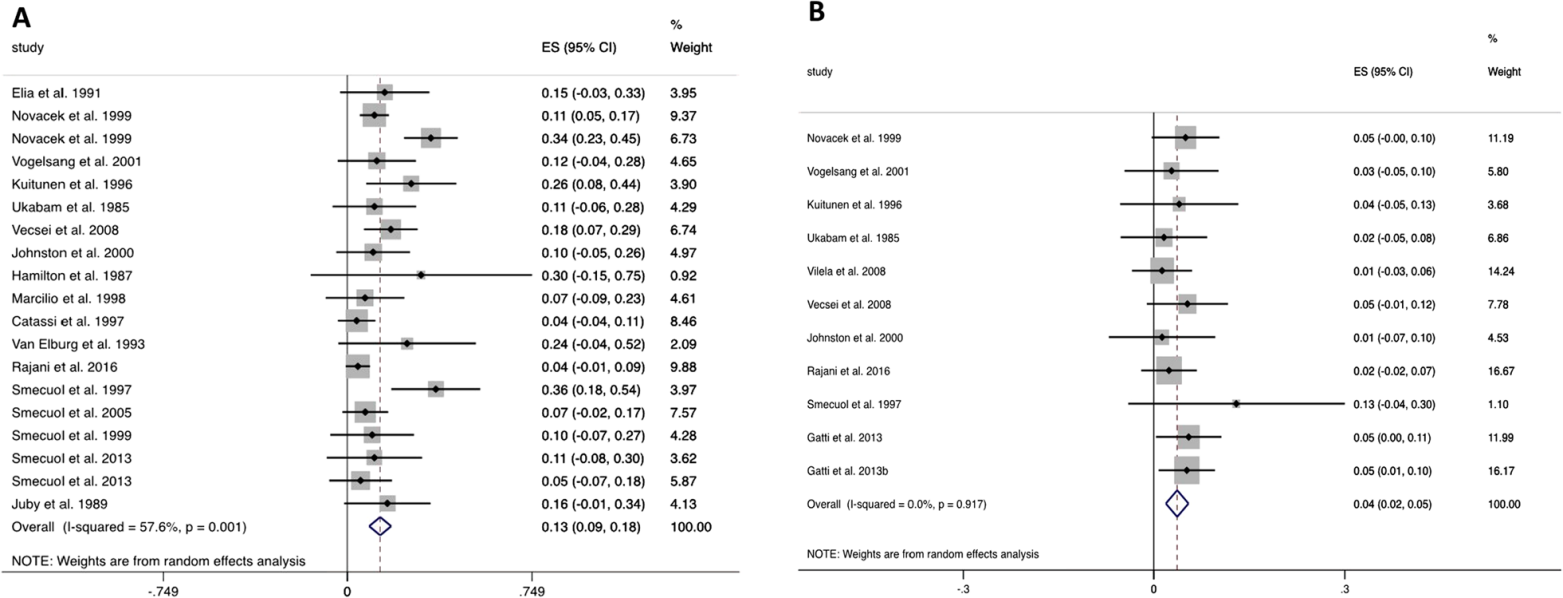
Forest plots showing pooled LMR (weighted mean) values in coeliac disease. **A** Untreated coeliac disease. **B** Treated coeliac disease

**Fig. 3:**
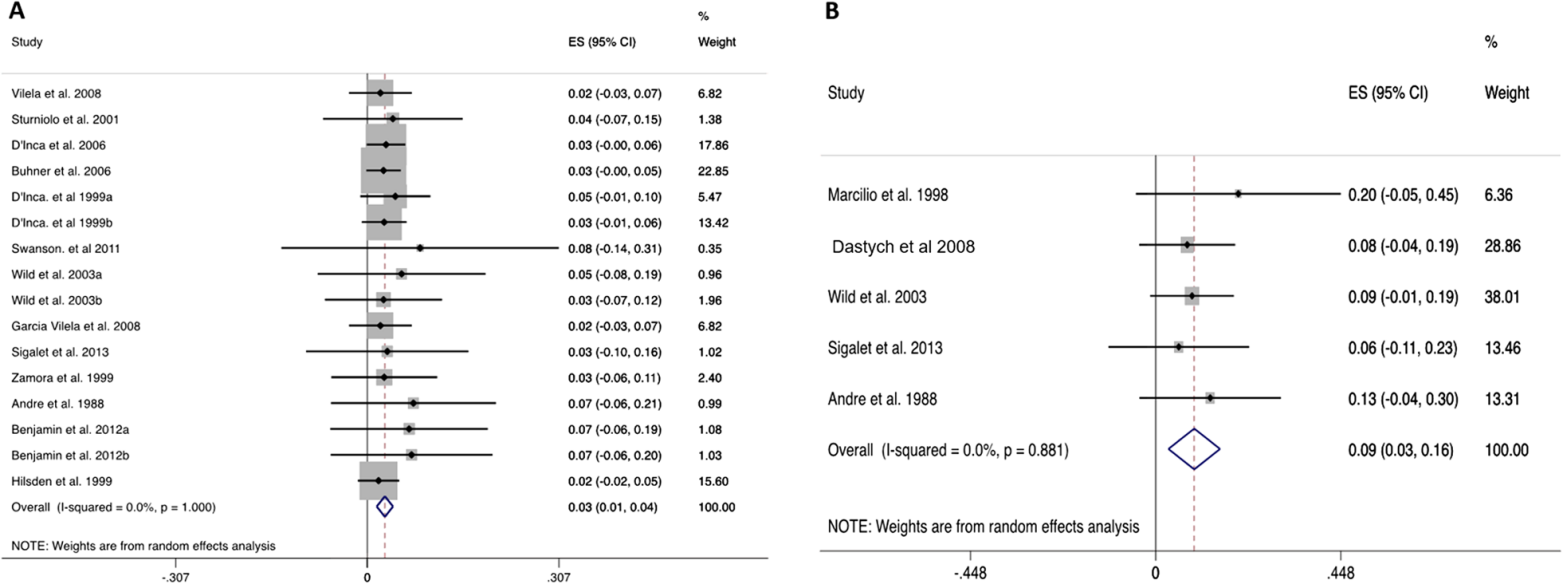
Forest plots showing pooled LMR values (weighted mean) in Crohn’s disease. **A** Inactive Crohn’s disease. **B** Active Crohn’s disease

Pooled analysis of gut permeability results in healthy subjects analysed in 24 studies (Fig. 1) revealed a LMR value of 0.014 (95% CI: 0.006 to 0.022). In untreated and treated coeliac patients (Fig. 2A, B), the pooled LMR values were 0.133 (95% CI: 0.089 to 0.178) and 0.037 (95% CI: 0.019 to 0.055) respectively. In inactive Crohn’s disease (Fig. 3A), the pooled LMR 0.028 (95% CI: 0.015 to 0.041), while in active Crohn’s disease (Fig. 3B), the pooled LMR was 0.093 (95% CI: 0.031 to 0.156).

#### LMR comparisons in coeliac disease

The SMD and WMD in LMR between healthy controls and treated coeliac disease (4 studies) was 0.409 (95% CI: 0.034 to 0.783, *p* = 0.032, Additional file 1: Fig S3A) and 0.009 (95% CI 0.003 to 0.014, *p* = 0.001, Additional file 1: Fig S3B) respectively. The SMD and WMD in LMR between healthy controls and patients with untreated coeliac disease (9 studies) were calculated as 1.362 (95% CI: 0.740 to 1.984, *p* < 0.001, Additional file 1: Fig S3C) and 0.090 (95% CI: 0.054 to 0.126, *p* < 0.001, Additional file 1: Fig S3D) respectively. The results exhibited high heterogeneity for comparison between healthy controls and untreated coeliac (I^2^ = 84.8%), but this was not the case not for the comparison between healthy controls and treated coeliac (I^2^ = 31.7%). The SMD and WMD in LMR between treated and untreated coeliac disease (6 studies) were 0.722 (95% CI: 0.286 to 1.157, *p* = 0.001, Additional file 1: Fig S3E) and 0.101 (95% CI: 0.040 to 0.162, *p* = 0.001, Additional file 1: Fig S3F) respectively, and the results were found to be heterogenous (I^2^ = 72.9%).

#### LMR comparisons in Crohn’s disease

11 studies were included in comparisons of LMR values in Crohn’s disease. 9 studies were included in the pooled random effects analysis of LMR in healthy controls versus inactive Crohn’s disease, revealing a SMD and WMD of 1.265 (95% CI: 0.845 to 1.686, *p* < 0.001, Additional file 1: Fig S4A) and 0.017 (95% CI: 0.012 to 0.022, *p* < 0.001, Additional file 1: Fig S4B) respectively. 5 studies comparing healthy controls and active Crohn’s disease were identified, showing a SMD and WMD in LMR of 2.868 (95% CI: 2.112 to 3.623, *p* < 0.001, Additional file 1: Fig S4C) and 0.078 (95% CI: 0.049 to 0.107, *p* < 0.001, Additional file 1: Fig S4D) respectively. High heterogeneity was observed in both comparisons (I^2^ values of 85.8% and 71.8% respectively). 3 studies were included in the comparison of active and inactive Crohn’s disease, showing a SMD and WMD of 1.429 (95% CI: 0.580 to 2.278, *p* = 0.001, Additional file 1: Fig S4E) and 0.042 (95% CI: 0.021 to 0.063, *p* < 0.001, Additional file 1: Fig S4F) respectively, and the results were found to be heterogenous (I^2^ = 74%).

### Subgroup comparisons using different solutes

We also sought to examine if there were any differences in the results obtained when using different lactulose:mannitol ratios in the solutes given to patients during the L:M test. The solutes used in the studies cited here can be broadly divided into ratios of 5:2 (5 parts lactulose to 2 parts mannitol) and 2:1 (2 parts lactulose to 1 part mannitol).

#### 5:2 solute used in Crohn’s and coeliac disease

Using 5:2 solutes, the SMD and WMD in LMR between healthy controls and untreated coeliac disease (6 studies) were found to be 1.495 (95% CI: 0.549 to 2.441, *p* = 0.002) and 0.072 (95% CI: 0.033 to 0.11, *p* < 0.001) respectively. This was associated with high heterogeneity (I^2^ = 89.6%). In treated versus untreated coeliac disease (2 studies), the SMD and WMD in LMR were 0.401 (95% CI: − 0.003 to 0.806, *p* = 0.052) and 0.107 (95% CI: − 0.097 to 0.311, *p* = 0.305) respectively. This was associated with low heterogeneity (I^2^ = 25.6%). Only one study was found in which healthy controls were compared with treated coeliac patients using 5:2 solutes and hence no analysis was done for this.

In studies comparing patients with Crohn’s disease and healthy subjects, 4 studies were found using 5:2 solutes. The SMD and WMD in LMR between inactive Crohn’s disease and healthy controls (2 studies) were 0.284 (95% CI: − 0.273 to 0.841, *p* = 0.318) and 0.003 (95% CI: − 0.005 to 0.011, *p* = 0.486) respectively. The heterogeneity of results was low (I^2^ = 0%). The analysis of SMD and WMD in LMR between active Crohn’s disease and healthy controls (2 studies) revealed a difference 2.941 (95% CI: 0.156 to 5.725, *p* = 0.038) and 0.099 (95% CI: − 0.048 to 0.246, *p* = 0.186) respectively, and the results were highly heterogeneous (I^2^ = 91.3%). Only 1 study was found where inactive versus active Crohn’s was compared using the 5:2 solute and hence no analysis was done for this.

#### 2:1 solute used in Crohn’s and coeliac disease

9 studies investigated the difference in LMR between healthy controls and coeliac disease using the 2:1 solute. Comparing healthy controls against untreated coeliac disease (4 studies), the SMD and WMD in LMR were calculated as 1.737 (95% CI: 0.701 to 2.773, *p* = 0.001) and 0.103 (95% CI: 0.038 to 0.167, *p* = 0.002) respectively. This was associated with high heterogeneity (I^2^ = 85.9%). Comparing LMR in healthy controls against treated coeliac disease in 3 studies revealed a SMD and WMD of 0.604 (95% CI: 0.212 to 0.997, *p* = 0.003) and 0.009 (95% CI: 0.004–0.014, *p* < 0.0001) respectively, which was associated with low heterogeneity (I^2^ = 0%). Analysis of SMD and WMD in LMR between treated and untreated coeliac patients (4 studies) revealed a difference of 0.992 (95% CI: 0.200 to 1.645, *p* = 0.012) and 0.103 (95% CI 0.061 to 0.144, *p* < 0.001) respectively, and this was associated with high heterogeneity (I^2^ = 81.3%).

9 studies performed comparisons of LMR in patients with Crohn’s disease using the 2:1 solute. Analysis of 6 studies comparing SMD and WMD in healthy controls and inactive Crohn’s disease revealed a change of 1.442 (95% CI: 0.944 to 1.941, *p* < 0.001) and 0.018 (95% CI: 0.014–0.023, *p* < 0.001) respectively which was associated with high heterogeneity (I^2^ = 88.3%). 3 studies were found comparing healthy controls and active Crohn’s disease, and analysis revealed SMD and WMD in LMR of 3.312 (95% CI: 2.257 to 4.366, *p* < 0.001) and 0.089 (95% CI: 0.056 to 0.121, *p* < 0.001) with high heterogeneity (I^2^ = 73%). Only 1 study was found where inactive versus active Crohn’s was compared using the 2:1 solute and hence no analysis was done for this.

### Sensitivity and specificity analysis

We analysed the sensitivity and specificity based on the available data in the papers included in our review. 4 studies reported diagnostic accuracies for the L:M test in screening for coeliac disease. The sensitivity and specificity data are presented in Additional file 1: Table S4. Pooled specificity (Additional file 1: Fig S5A) was calculated as 0.700 (95% CI: 0.551–0.849), and pooled sensitivity (Additional file 1: Fig S5B) was calculated as 0.829 (95% CI: 0.682–0.976). However, the overall heterogeneity for sensitivity and specificity was high (I^2^ = 94.3% and 78.4% respectively).

Sensitivity and specificity values for coeliac disease were also calculated based on a SROC curve (Additional file 1: Fig S5C). The pooled, weighted AUC was calculated as 0.88 (95% CI: 0.85–0.91). Based on the SROC graph, the estimated positive likelihood ratio was 4.0 (95% CI: 1.5–10.6) and the estimated negative likelihood ratio was 0.15 (95% CI: 0.04–0.6). The estimated diagnostic odds ratio was 27 (95% CI: 4–194). The estimated sensitivity and specificity from the SROC graph were 0.89 (95% CI: 0.62–0.97) and 0.78 (95% CI: 0.51–0.92) respectively, in agreement with the pooled results reported above.

Sensitivity and specificity analysis were not performed for Crohn’s disease due to paucity of data in the studies included in our meta-analysis.

### Risk of bias assessment

In 19 studies where the Newcastle Ottawa Score was used to assess bias, 7 studies had low risk, and 12 had moderate risk. Risk of bias was assessed using the Cochrane Risk of bias tool in 4 RCT studies, and we found that 1 studies had low risk of bias, 2 had moderate risk of bias and 1 study had high risk of bias. 14 studies were assessed using the ROBINS-I tool, and we found that 1 study had low risk of bias, 9 studies had moderate risk of bias and 4 studies had serious risk of bias. The detailed assessments of bias in each domain using the NOS and ROBINS-1 are presented in the Additional file 1: Tables B1-B4, Fig S6-S9.

## Discussion

Overall, this meta-analysis demonstrates that despite considerable heterogeneity in the data, there are significant differences in LMR between healthy subjects and patients with either coeliac or Crohn’s disease. There are also significant differences in LMR between treated and untreated coeliac, and active compared to inactive Crohn’s disease.

These results hold true even when different L:M solute ratios are used. Altogether, there were 18 studies that used a 2:1 ratio of lactulose to mannitol, and 13 studies that used a 5:2 ratio. While no previous studies have performed direct comparisons of the data obtained using different solute ratios, we found that standard mean differences in LMR were larger when using the 2:1 ratio than the 5:2 (although high heterogeneity in the data means that this observation should be taken with caution, and statistical significance was not observed). However, the numbers in each subgroup were small, which may explain the mixed significances and heterogeneities observed. Nevertheless, this raises the possibility that the heterogenous nature of our results could be attributed to the different L:M solute ratios used, along with other factors such as assay method, time of fasting before the solute is administered, and urine collection times.

Musa et al. found no significant difference in LMR when comparing prolonged urine collection time (5 h) with urine collected over a 2 h period [54]. In the same study, they also found no significant differences when using two different analysis methods: high-performance anion exchange chromatography with derivatization-free, pulsed amperometric detection (HPAE-PAD); and liquid chromatography with tandem mass spectrometry (LC-MSMS) [54]. This concurred with results from Akram’s earlier study regarding urine collection, which found no significant differences in LMR when urine was collected over 2 and 6 h [55]. Camilleri et al., on the other hand, found that LMR based on urine collections over 8–24 h were significantly higher than those for collections times of 0–2 h [6]. Interestingly, a study by Sequeira and colleagues suggested that differences in temporal patterns of excretion of lactulose and mannitol can be minimised if the urine collection period is restricted to 2½-4 h after solute ingestion [4].

In relation to analysis platforms used for quantification of urinary lactulose and mannitol, Lee et al. found that LC-MSMS provides more accurate measurements than HPAE-PAD [56]. They subsequently recommended the former to be used in L:M studies. Nonetheless, current evidence surrounding the variable protocols used in L:M studies (e.g. in terms of the analysis platforms, solute ratios and urinary collection times used) is mixed, and more studies are required to elucidate the optimal method for performing this test.

Despite the variability and heterogeneity observed, we found significant differences in LMR between healthy controls and untreated coeliac disease. Patients with active coeliac disease are known to have flat mucosa, increased villous height, and increased paracellular permeability due to wider tissue junction pores and release of pro-inflammatory cytokines [57–59]. Gluten is thought to activate zonulin signalling, which opens up the tight junctions, causing increased paracellular permeability [60]. The role of gut permeability in the pathogenesis of coeliac disease is currently poorly understood, but it is thought that it might act to self-sustain the inflammatory response and perpetuate a vicious cycle [61]. Regardless, the increased permeability leads to an increase in lactulose excretion into urine, resulting in significantly higher LMR values than those observed in healthy subjects.

Differences in LMR between treated and untreated coeliac disease were also observed and found to be significant, with the change in LMR observed across all coeliac studies included in this review. These changes need to be analysed with caution, however, as the results were heterogenous and the 95% confidence interval was fairly wide (0.029–0.218).

There were also significant differences between the LMR values observed in treated coeliac disease and healthy controls, implying that it may take some time before mucosal integrity returns to baseline. A study by Cummins et al. showed (via the L:R test) that gut permeability improves after 2 months on a gluten free diet (GFD), but that it takes up to 6 months before villous recovery is observed [62]. Duerksen and colleagues demonstrated that more than 80% of coeliac patients on GFD for at least a year exhibited reduced gut permeability, although permeability only returned to normal levels in 48% of patients (10/21) [63]. Rajani et al. (one of the papers included in this analysis) reported no significant difference in LMR between healthy controls and coeliac patients who followed a GFD for a year [44]. Similarly, Vogelsang et al. (another paper included in this analysis) also reported no significant difference in LMR when comparing healthy subjects against coeliac patients who had a median of 44 months on a GFD [35]. However, LMR values were significantly different (healthy vs. treated coeliac) in the studies published by Vilela et al. (1 year of GFD) [19] and Ukabam et al. (5–8 months of GFD) [38]. Thus, current evidence points towards the role of a GFD in improving gut permeability and restoring gut integrity after more than 12 months.

Another important finding in this review is the presence of significant differences in LMR between healthy controls and both active and inactive Crohn’s disease. Moreover, significant differences were also observed between active and inactive Crohn’s patients. While the pathogenesis of Crohn’s disease is multifactorial and the link to gut permeability is still not well understood, a study in 2019 using three-dimensional tissue culture models demonstrated that epithelial barrier dysfunction may be caused by Tumour Necrosis Factor (TNF)-α induced tight junction modulation and involvement of the c-Jun N-terminal protein kinase mitogen-activated protein kinases (JNK MAPK) signalling pathway [64]. Furthermore, altered gut permeability is surmised to be present at the early stages of disease, as increased paracellular permeability was found even in patients with quiescent IBD where endoscopic activity was absent [65]. Techniques other than the L:M test have also been used to assess gut permeability in Crohn’s disease. For example, in a recent study, a moderate positive correlation was found between excreted Chromium-52 labelled ethylenediamine tetraacetic acid (^52^Cr-EDTA) and faecal calprotectin levels (a known marker of gut permeability) in Crohn’s patients [66]. Similarly, the use of zonulin [67], Ussing chambers [68], and PEG tests [69] have also demonstrated increases in gut permeability in Crohn’s disease. Thus, our findings are in agreement with the above studies and provide further evidence for the importance of gut permeability in Crohn’s disease.

Interestingly, the differences in LMR between controls, active and inactive Crohn’s disease were smaller than those observed for untreated coeliac disease. This may indicate that the breakdown in epithelial barrier function is more pronounced in coeliac disease than it is in Crohn’s. Despite this, there was again significant heterogeneity in the difference values observed between control and active Crohn’s disease, and between inactive and active Crohn’s disease. This further indicates that observations made with the L:M test need to be taken with caution.

There are many advantages of the L:M test in measuring gut permeability. It is easy to perform, inexpensive and non-invasive. Our data also suggests that this test is associated with high sensitivity, making it a useful tool in screening for coeliac disease. (Sensitivity and specificity analysis was not performed for Crohn’s disease as the necessary data was not available in the papers included in our review). The L:M test was the method of choice in the MAL-ED study, which investigated the link between gut permeability and environmental enteropathy in children across 8 countries [70]. However, there is great variability in how the test is performed and our meta-analysis has revealed considerable heterogeneity in the results obtained. Interestingly, Ordiz et al.—who conducted the L:M test in 1669 rural Malawian children—surmised that the strong direct correlation between percentage lactulose and percentage mannitol excretion does not support the use of mannitol as a normalising factor for lactulose, and that using percentage lactulose excretion alone actually yields more information about gut integrity than LMR [71]. In addition, L:M measurements performed by Camilleri et al. indicated that LMR at 0–2 h may in part reflect colonic permeability and not exclusively small bowel permeability. They have hence recommended that measurements of small bowel permeability using urine collected in the L:M test over 0–6 h should be treated with caution [6].

While there are clear limitations to the L:M test, the quantification of gut permeability in coeliac and Crohn’s disease reported here highlights a potential route for clinicians to better understand other gastrointestinal conditions where current diagnostics can be improved. For example, as larger changes in LMR were obtained in coeliac disease than in the Crohn’s disease (relative to healthy controls), this implies the possibility to stratify patients according to their gut permeability. Similarly, as differences were observed between treated and untreated patients, this suggests an opportunity to monitor for signs of relapse in a non-invasive manner (i.e. without the need for endoscopy). Thus, quantifying gut permeability may provide an avenue for the practising clinician to better assess patients with FGDs. Indeed, there is promise in utilising gut permeability values to improve management of this complex group of patients in either the primary care setting or the gastroenterology clinic. Nonetheless, we stress that the results of our meta-analysis do not necessarily suggest that the L:M test is currently suitable for this purpose. The high heterogeneity observed across groups and datasets means that it is unlikely that the L:M test will find widespread clinical use in its current form (and indeed explains why it has not done so to date). Hence, if assessment of gut permeability is to find widespread use in the diagnosis of FGDs, Crohn’s, coeliac or other conditions then it is highly likely that improved diagnostic tools/methods (or at the very least improved protocols for deployment of the L:M test) will be required.

### Limitations

The main limitation of this meta-analysis is the heterogeneity of our results, which is likely to be explained by the variations in how the L:M test was performed (and by physiological variations across individuals). A list of protocol variations that may have caused the heterogeneity is presented in Additional file 1: Table S3. It is also difficult to directly assess the results of the two different solutes used due to the heterogeneity in most of these comparisons. In one of the studies [18], both 5:2 and 2:1 lactulose: mannitol ratios in the solutes were given to patients during the L:M test, making it more difficult to compare the differences in LMR results between the two solutes. Furthermore, most studies that were available and included in the meta-analysis were at significant risk of bias. There are also variations in the way sensitivity and specificity were measured. As shown in Additional file 1: Table S4, due to the heterogenous nature of the L:M test, different cut-off point values were used in included studies for diagnosis of disease. There were also not many studies that assessed the value of the L:M test as a screening measure in coeliac or Crohn’s disease. In terms of data analysis, DTA was only applicable on one subset of the data (Additional file 1: Fig S5). Overall, there was insufficient data available to perform a diagnostic accuracy meta-analysis.

Another limitation in this meta-analysis is in the variability in how active or inactive Crohn’s was defined as evidenced in Additional file 1: Table S1. Crucially, however, most of these limitations are inherent to the L:M test itself. Thus, the fact that our results were heterogeneous highlights these important limitations to the L:M test and reveals that improvements are required if it is to be more widely used for clinical assessment of gut permeability.

Overall, this review has quantified the diagnostic value of the L:M test and reported the LMR values in healthy subjects, treated and untreated coeliac disease, and inactive and active Crohn’s disease. In addition, it provides a quantification of the heterogeneity in LMR values observed in these disease states. Our analysis demonstrates that there is potential value in measuring gut permeability in both Crohn’s and coeliac disease, and it provides an insight into the role of gut permeability in the pathogenesis of both conditions. Importantly, however, it also highlights the limitations in the L:M test and the need for both improved protocols and alternative diagnostic tools.

## Conclusion

Gut permeability is significantly impaired in untreated coeliac and Crohn’s disease. Gut barrier function is then recovered as patients are treated appropriately. These changes can be observed using the L:M test and this meta-analysis reports pooled LMR values in both diseases. While the L:M test can provide good diagnostic accuracy and offers some insight into gut permeability, it is limited by the lack of standardisation and the length of time required to conduct the test. Thus, if the L:M test is to find wider clinical use, then an optimised, standardised protocol needs to be determined and used. Even if this is achieved, the limited use of the L:M test may persist due to physiological variations between subjects and limitations in the accuracy of the test caused by changes in the permeation of mannitol. As such, there is a strong case for the development of new diagnostic tools that can provide faster, more accurate and more reliable quantification of gut permeability in a minimally or non-invasive manner. Such devices would have potential in monitoring progression/resolution of diseases such as coeliac and Crohn’s, and in identifying patients at risk of relapse. This is particularly true in the paediatric population, where difficulties when using the L:M test were experienced because of the logistics associated with the test [9, 54]. There is now an improved understanding of the increasing use of gut permeability analysis in patient care, and as a result, there is a concomitant need for robust evidence to develop this field for its next stage of healthcare innovation.

## Supplementary Information

**Additional file 1**: **Appendix 1**: Search Strategy for gut permeability and lactulose mannitol test in disease in Medline. **Appendix 2**: Search Strategy for gut permeability and lactulose mannitol test in disease in Embase. **Appendix 3**: Search Strategy for gut permeability and lactulose mannitol test in disease in Cochrane. **Figure S1**: PRISMA 2009 Flow diagram for coeliac disease. **Figure S2**: PRISMA 2009 Flow diagram for Crohn’s disease. **Figure S3A**: Standard Mean Difference (SMD) in LMR between treated coeliac disease and healthy controls. **Figure S3B**: Weighted Mean Difference (WMD) in LMR between treated coeliac disease and healthy controls. **Figure S3C**: Standard Mean Difference (SMD) in LMR between untreated coeliac disease and healthy controls. **Figure S3D**: Weighted Mean Difference (WMD) in LMR between untreated coeliac disease and healthy controls. **Figure S3E**: Standard Mean Difference (SMD) in LMR between untreated and treated coeliac disease. **Figure S3F**: Weighted Mean Difference (WMD) in LMR between untreated and treated coeliac disease. **Figure S4A**: Standard Mean Difference (SMD) in LMR between healthy controls and inactive Crohn's disease. **Figure S4B**: Weighted Mean Difference (WMD) in LMR between healthy controls and inactive Crohn's disease. **Figure S4C**: Standard Mean Difference (SMD) in LMR between active Crohn's disease and healthy controls. **Figure S4D**: Weighted Mean Difference (WMD) in LMR between active Crohn's disease and healthy controls. **Figure S4E**: Standard Mean Difference (SMD) in LMR between active and inactive Crohn's disease. **Figure S4F**: Weighted Mean Difference (WMD) in LMR between active and inactive Crohn's disease. **Figure S5**: Sensitivity and specificity of the L:M test in coeliac disease. **Figure S6**: Risk of bias for each risk of bias item in RCT studies. **Figure S7**: Risk of bias assessments presented per risk of bias domain in RCT studies. **Figure S8**: Risk of bias for each risk of bias item in non-randomised and cohort studies. **Figure S9**: Risk of bias assessments presented per risk of bias domain in non-randomised and cohort studies. **Table S1**: Summary of studies of gut permeability in Crohn’s disease. **Table S2**: Summary of studies of gut permeability in coeliac disease. **Table S3**: Sources of variability for the studies included in the meta-analysis. **Table S4**: Studies depicting sensitivity and specificity of LMR in screening for coeliac disease. **Table B1**: Newcastle Ottawa Score Assessing Risk of Bias for Case Control Studies. **Table B2**: Newcastle Ottawa Score Assessing Risk of Bias for Cross Sectional Studies. **Table B3**: Risk of Bias for Randomised Control Trials (RCT) using the Cochrane Risk of Bias Tool. **Table B4**: Risk of Bias in non-randomised trials and cohort studies using the ROBINS-I score.

## Data Availability

The dataset used and/or analysed during the current study are available from the corresponding author on reasonable request.

## Abbreviations

GI: Gastrointestinal
IBD: Inflammatory bowel disease
L:M Test: Lactulose mannitol test
L:R Test: Lactulose rhamnose test
PEG: Polyethylene glycol
LPS: Lipopolysaccharide
FGD: Functional gut disorder
Cr-EDTA: Chromium-51 labelled ethylenediamine tetraacetic acid
LMR: Lactulose mannitol ratio
PRISMA: Preferred reporting items for systematic reviews and meta-analyses
PROSPERO: International prospective register of systematic reviews
MEDLINE: Medical literature analysis and retrieval system online
ROBINS-I tool: Risk of bias in non-randomised studies of interventions tool
NOS: Newcastle Ottawa score
SMD: Standard mean difference
WMD: Weighted mean difference
CI: Confidence interval
SROC model: Summary receiver operating characteristic model
AUC: Area under the curve
DTA: Diagnostic test accuracy
IQR: Inter-quartile range
SEM: Standard error of mean
HPAE-PAD: High-performance anion exchange chromatography with derivatization-free, pulsed amperometric detection
LC-MSMS: Liquid chromatography with tandem mass spectrometry
GFD: Gluten-free diet
TNF: Tumour necrosis factor
JNK MAPK: C-Jun N-terminal protein kinase mitogen-activated protein kinase
^52^Cr-EDTA: Chromium-52 labelled ethylenediamine tetraacetic acid
MAL-ED Study: Etiology, Risk Factors and Interactions of Enteric Infections and Malnutrition and the Consequences for Child Health and Development Study
