# Supplementary material for: A case for improved assessment of gut permeability: a meta-analysis quantifying the lactulose:mannitol ratio in coeliac and Crohn’s disease
Source: BMC Gastroenterol. 2022 Jan 10;22:16. doi: 10.1186/s12876-021-02082-z (PMC8751358; doi:10.1186/s12876-021-02082-z)
Supplement: Supplementary file 1 — Additional file 1: Appendix 1: Search Strategy for gut permeability and lactulose mannitol test in disease in Medline. Appendix 2: Search Strategy for gut permeability and lactulose mannitol test in disease in Embase. Appendix 3: Search Strategy for gut permeability and lactulose mannitol test in disease in Cochrane. Figure S1: PRISMA 2009 Flow diagram for coeliac disease. Figure S2: PRISMA 2009 Flow diagram for Crohn’s disease. Figure S3A: Standard Mean Difference (SMD) in LMR between treated coeliac disease and healthy controls. Figure S3B: Weighted Mean Difference (WMD) in LMR between treated coeliac disease and healthy controls. Figure S3C: Standard Mean Difference (SMD) in LMR between untreated coeliac disease and healthy controls. Figure S3D: Weighted Mean Difference (WMD) in LMR between untreated coeliac disease and healthy controls. Figure S3E: Standard Mean Difference (SMD) in LMR between untreated and treated coeliac disease. Figure S3F: Weighted Mean Difference (WMD) in LMR between untreated and treated coeliac disease. Figure S4A: Standard Mean Difference (SMD) in LMR between healthy controls and inactive Crohn's disease. Figure S4B: Weighted Mean Difference (WMD) in LMR between healthy controls and inactive Crohn's disease. Figure S4C: Standard Mean Difference (SMD) in LMR between active Crohn's disease and healthy controls. Figure S4D: Weighted Mean Difference (WMD) in LMR between active Crohn's disease and healthy controls. Figure S4E: Standard Mean Difference (SMD) in LMR between active and inactive Crohn's disease. Figure S4F: Weighted Mean Difference (WMD) in LMR between active and inactive Crohn's disease. Figure S5: Sensitivity and specificity of the L:M test in coeliac disease. Figure S6: Risk of bias for each risk of bias item in RCT studies. Figure S7: Risk of bias assessments presented per risk of bias domain in RCT studies. Figure S8: Risk of bias for each risk of bias item in non-randomised and cohort studies. Figure S9: Risk of bias assess [file 12876_2021_2082_MOESM1_ESM.pdf]

SUPPLEMENTARY INFORMATION

A case for improved assessment of gut permeability – a meta-analysis quantifying the lactulose:mannitol ratio in coeliac and Crohn's disease

Jonathan Gan[1*], Scarlet Nazarian[1], Julian Teare[1], Ara Darzi[1], Hutan Ashrafian[1†], Alex J. Thompson[1,2†]

[1]Department of Surgery & Cancer, 10th Floor, Queen Elizabeth the Queen Mother Wing, St Mary's Hospital, Imperial College London, South Wharf Road, London, W2 1NY, UK
[2]Hamlyn Centre for Robotic Surgery, Level 3 Paterson Building, St Mary's Hospital, Imperial College London, South Wharf Road, London, W2 1NY, UK

*Corresponding author: Jonathan Gan, Institute of Global Health Innovation, Imperial College London, email: jgwei@ic.ac.uk

†These authors contributed equally to this work

This PDF file contains:

- Appendices 1-3 (Search Strategies)
- Supplementary Figures S1-S9
- Supplementary Tables S1-S4
- Tables B1-B4

Search Strategies

The following search strategies were used to identify studies using the lactulose mannitol test in measuring gut permeability in disease. Subsequently studies in Crohn's and coeliac disease were manually identified from within these groups.

Appendix 1: Search Strategy for gut permeability and lactulose mannitol test in disease in Medline

Database: Ovid MEDLINE(R) and Epub Ahead of Print, In-Process & Other Non-Indexed Citations and Daily <1946 to December 16, 2019>
Search Strategy:
----
1 Permeability/ (34317)
2 Permeability.mp. (158534)
3 (leaky or leakiness).mp. (7152)
4 or/1-3 (164265)
5 lactulose mannitol.mp. (467)
6 4 and 5 (429)
7 exp Animals/ (22810673)
8 Humans/ (18159647)
9 7 not 8 (4651026)
10 6 not 9 (381)
11 rat$1.ti. (951083)
12 10 not 11 (377)

Appendix 2: Search Strategy for gut permeability and lactulose mannitol test in disease in Embase

Database: Embase <1974 to 2019 December 16>
Search Strategy:
----
1 Permeability/ (25617)
2 intestine mucosa permeability/ (6768)
3 Permeability.mp. (199965)
4 (leaky or leakiness).mp. (8451)
5 or/1-4 (206514)
6 lactulose mannitol.mp. (699)
7 5 and 6 (649)
8 exp animal/ (24871330)
9 exp human/ (20320781)
10 8 not 9 (4550549)
11 7 not 10 (569)
12 rat$1.ti. (1027775)
13 11 not 12 (569)

Appendix 3: Search Strategy for gut permeability and lactulose mannitol test in disease in Cochrane

ID	Search	Hits
#1	MeSH descriptor: [Permeability] this term only	373
#2	permeability	3098
#3	leaky or leakiness	102
#4	#1 or #2 or #3	3164
#5	"lactulose mannitol"	230
#6	#4 and #5	191

#7	MeSH descriptor: [Animals] explode all trees	15676
#8	MeSH descriptor: [Humans] this term only	8475
#9	#7 not #8	7201
#10	#6 not #9	190

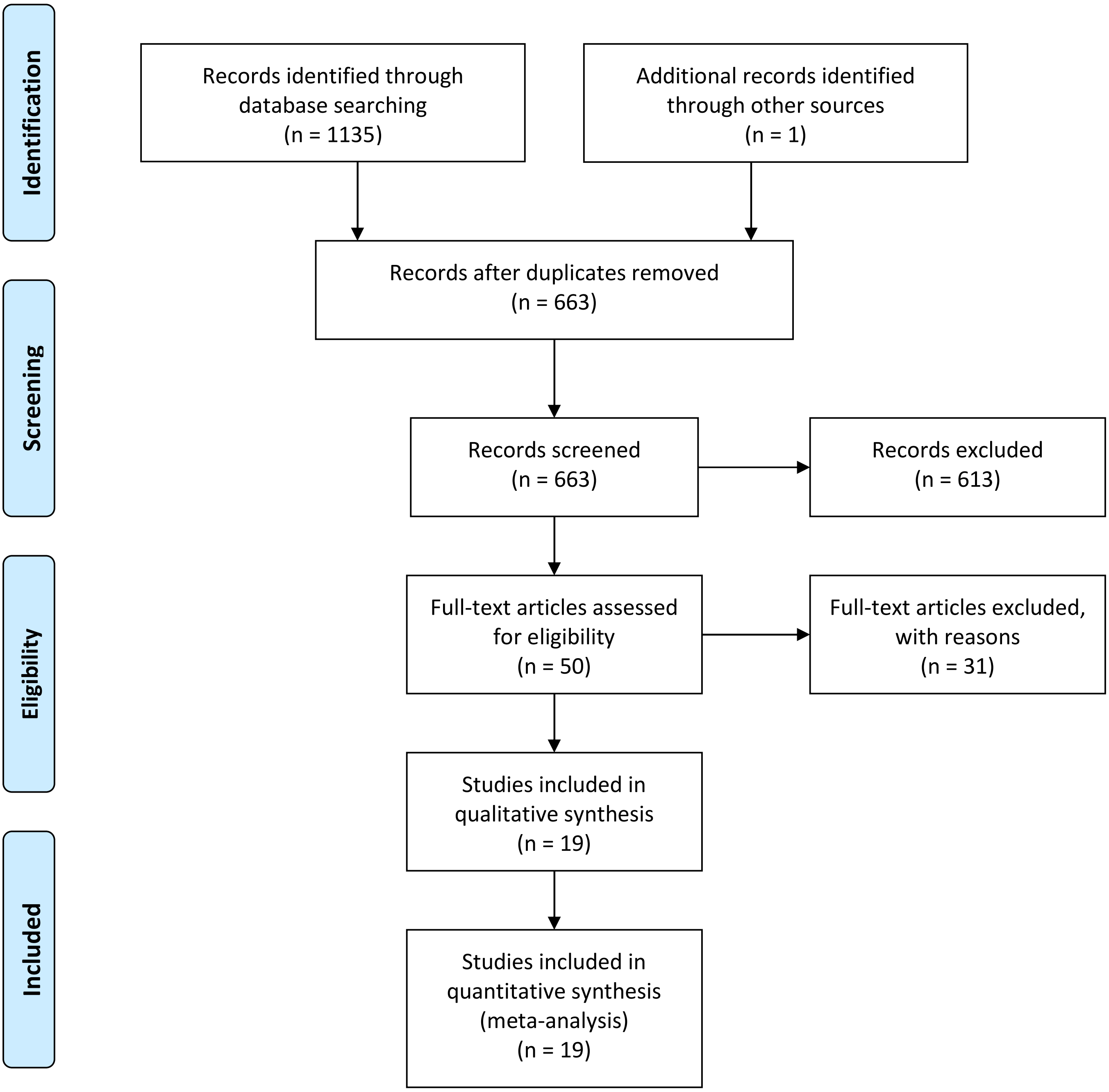

Fig. S1. PRISMA 2009 Flow diagram for coeliac disease.

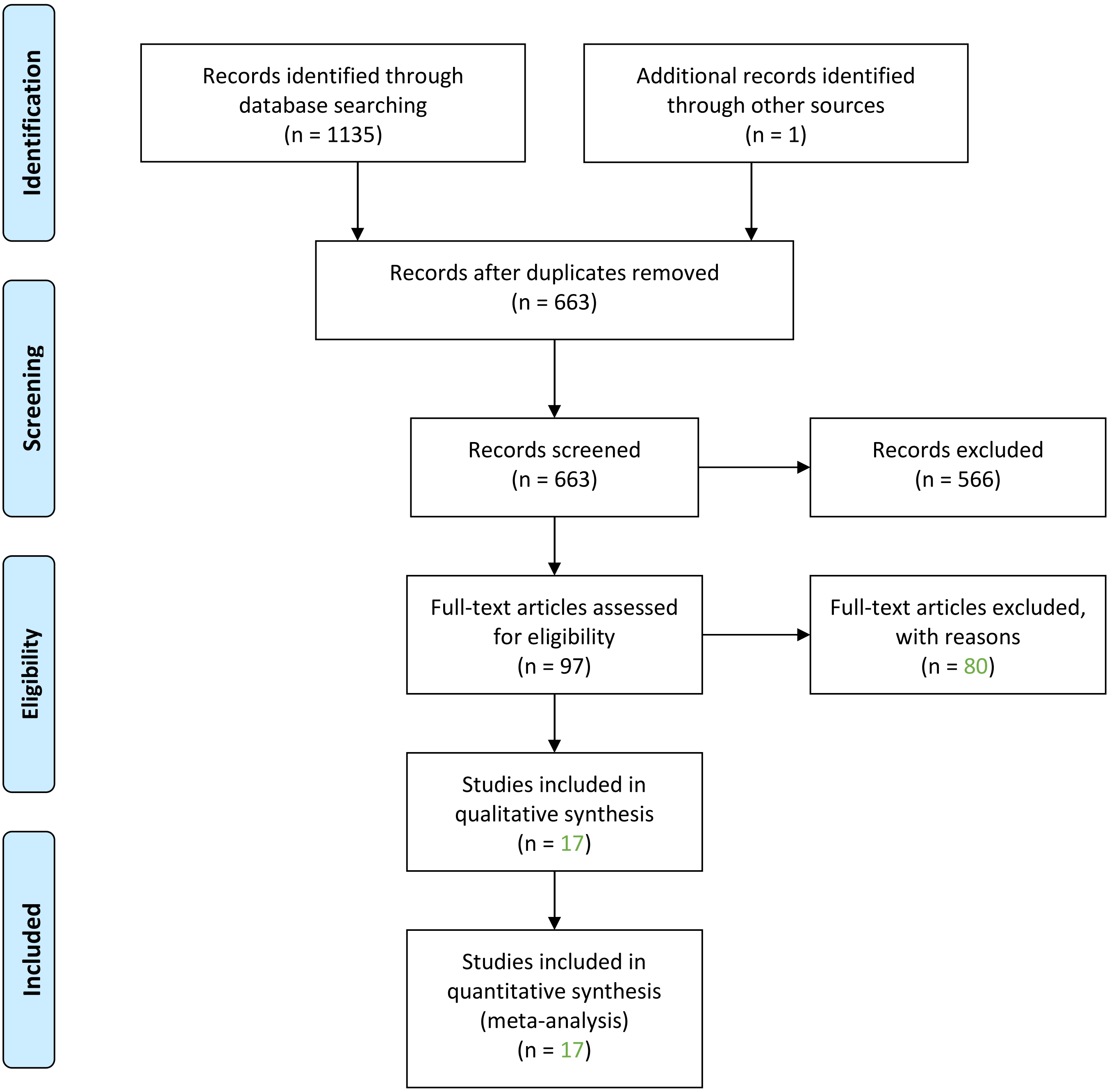

Fig. S2. PRISMA 2009 Flow diagram for Crohn’s disease.

Study	Study type	Method of diagnosis	Definition of active Crohn's/relapse	Definition of inactive Crohn's/remission	Number of people who had surgery	Management	Controls	Patient population compared	Recruitment setting of patients with Crohn's disease	Time of urine collection	L:M solute ratio	NBM period pre-solute ingestion	Method of analysis
Marsilio et al 1998	cross sectional	A	PCDAI>30		unk	unk	healthy	Patients with moderate/severe Crohn's iliocolitis, moderate/severe coeliac disease	Unk	6 hours	2:1 and 5:2	unk	HPLC
Vilela et al 2008	cross sectional	unk		CDAI<150	0%	A (32%), B (32%), A/B (6.5%), A/B and C (12.9%), D (3.2%), D/E (3.2%), F (9.6%)	healthy	Terminal ileum/ileocolic Crohn's disease in remission, coeliac patients after 1 year in GFD	Gastroenterology clinic in a university hospital	6 hours	2:1	>6 hours	HPLC
Sturniolo et al 2001	cohort	B+C+D+E		CDAI<150 for >3 months	0%	A	n/a	Ileal/ileocolic/colonic Crohn's disease in remission	Gastroenterology clinic in a university hospital	6 hours	2:1	>6 hours	enzymatic analysis
Dastych et al 2008	cross sectional	unk	CDAI 220-280		unk	unk	healthy	Active Crohn's in terminal ileum and colon, patients with liver cirrhosis	Unk	5 hours	2:1	>6 hours	enzymatic analysis
D'Inca et al 2006	cross sectional	B+C+D+E		Patients not manifesting clinical symptoms and not on steroids	14.80%	A/G (64%), no C	healthy	Inactive ileal/ileocolic/colonic Crohn's Disease, relatives of patients with Crohn's disease	Gastroenterology department in 2 hospitals	6 hours	2:1	>6 hours	unk
Buhner et al 2006	cross sectional	B+C+D+E		CDAI<150	0%	A (70%), C (39%), G (4%), F (16.4%)	healthy	Terminal ileal/ileocolic/colonic/UGI Crohn's disease in remission, first degree relatives and non-blood relatives	Gastroenterology department in a university hospital	5 hours	2:1	>6 hours	HPLC
D'Inca et al 1999	cohort	B+C+D+E	Combination of either CDAI>150, increase in CDAI value by 50 (or 75 if underwent resection) points and requiring steroids	CDAI<150 for more than 3 months	42.30%	A (33.8%)	n/a	Patients with Crohn's disease in remission	Gastroenterology clinic in a university hospital	6 hours	2:1	>6 hours	enzymatic analysis
Swanson et al 2011	case control	unk		CDAI<150	unk	A (66%), B (17%)	healthy	Patients with inactive Crohn's, inactive UC	Gastroenterology clinic in a university hospital	unk	2:1	>6 hours	gas chromatography
Wild et al 2003*	cohort	C+D+E	CDAI>200(active diseease) CDAI >150 and 100 points increase above baseline and/or need for surgery (relapse)	CDAI<150	16.70%	A+C	n/a	Patients with active ileal/ileocolic Crohn's before 10 weeks of tapering steroids treatment, same cohort of patients in remission post steroids treatment whose Crohn's disease eventually relapse, same cohort of patients in remission post steroids treatment who did not experience relapse	Gastroenterology clinic in a university hospital	7 hours	2:1	unk	HPLC
Garcia Vilela et al 2008	RCT	unk		CDAI<150	unk	A (35%), B (32.3%), A/B/C (12.9%), A/B (6.5%), D (3.2%), D/E (3.2%)	n/a	Terminal ileum/ileocolic Crohn's disease in remission (LMR measured before intervention with Saccharomyces boulardii)	Gastroenterology clinic in a university hospital	6 hours	2:1	>6 hours	HPLC
Sigalet et al 2013	cohort	A+D	CDAI 180-220	CDAI 60-120	0%	On A or C at admission. Then treated with B and/or C	healthy	Paediatric Crohn's patients with acute ileal disease before 8-12 weeks treatment with steroids and/or azathioprine, same cohort of paediatric Crohn's patients in remission after 8-12 weeks treatment with steroids and/or azathioprine	Admission from a Gastroenterology department clinic in a university hospital	12 hours	5:2	<6 hours	HPLC
Zamora et al 1999	case control	A	PCDAI>30		unk	unk, no A	healthy	Paediatric Crohn's patients in remission, parents and first-degree relatives	Gastroenterology clinic in a university hospital	unk	5:2	<6 hours	HPLC
Andre et al 1988	cross sectional	B+C+D+E	HBI>4	HBI 0-2	29.8%	unk	healthy	Crohn's patients in remission, patients with relapsing Crohn's, patients with mild Crohn's disease	Unk	5 hours	1:1	>6 hours	gas chromatography
Benjamin et al 2012	RCT	B+C+D+E		CDAI<150	13.30%	A+B+C+H	n/a	Upper GI/Ileocolonic/ colonic Crohn's in remission (LMR measured before intervention with whey protein or glutamine)	Gastroenterology and Inflammatory Bowel Disease (IBD) clinic in a university hospital	5 hours	5:2	>6 hours	enzymatic analysis
Hilsden et al 1996	case control	unk		CDAI<150	unk	unk	healthy	Crohn's disease in remission, first degree relatives	Gastroenterology clinic in a university hospital	7 hours	5:2	<6 hours	HPLC

Hilsden et al 1999	cohort	unk	CDAI>150 or increase by 100 points or if high dose steroids/surgery are needed	CDAI<150 for last 30 days	unk	A (24.6%), B (2%), Low dose C, (8.2%), I (61%)	n/a	Small or large bowel Crohn's disease in remission	Gastroenterology/surgical clinic in a university hospital	unk	5:2	unk	HPLC
Breslin et al 2001	case control	unk		CDAI<130	unk	unk	healthy	Spouses of patients with inactive Crohn's disease	Recruited from the community	unk	5:2	<6 hours	HPLC

A) CDAI/PCDAI criteria
B) Clinical (non-specific)
C) Radiological
D) Endoscopic
E) Histological / pathological
F) Modified HBI

A) Aminosalicylates
B) Azathioprine
C) Steroids
D) Thalidomide
E) Metronidazole
F) No meds
G) Sulphursalazine
H) Hematinics, multivitamins, calcium supplements
I)Surgery

Table S1. Summary of studies of gut permeability in Crohn's disease. Methods of diagnosis were separated into six categories (A-F) as defined at the base of the column. Management was separated into eight categories (A-H) as defined in the key at the base of the column. Abbreviations: RCT – Randomised Control Trial, CDAI – Crohn's Disease Activity Index, PCDAI—Paediatric Crohn's Disease Activity Index, unk – unknown, n/a – not applicable, GFD – Gluten Free Diet, UC – Ulcerative Colitis, CABG – Coronary Artery Bypass Graft surgery, UGI – Upper Gastrointestinal, HBI – Harvey Bradshaw Index, HPLC – High Performance Liquid Chromatography, NBM – nil by mouth. * In Wild et al 2003, 8 patients were excluded as they did not respond to steroid treatment, and the remaining 22 patients who experienced remission after steroid treatment were followed up.

Study	Study type	Method of diagnosis	Number of people who had surgery	Management	Controls	Patient population compared	Recruitment setting of coeliac patients	Time of urine collection	L:M solute ratio	NBM period pre-solute ingestion	Method of analysis
Marsilio et al 1998	cross sectional	A	unk	unk	healthy	Active Crohn's iliocolitis, moderate/severe coeliac disease	unk	6 hours	2:1 and 5:2	unk	enzymatic analysis
Novacek et al 1999	cohort	A+B	unk	A	n/a	Newly diagnosed coeliac with normal ALT/AST, newly diagnosed coeliac with abnormal ALT/AST	unk	5 hours	2:1	unk	HPLC
Vogelsang et al 2001	cross sectional	B	0%	B	Patients with non-specific gastroenterology symptoms	Partly treated coeliac disease, untreated coeliac disease and relatives of coeliac patients	Gastroenterology clinic in a university hospital	5 hours	2:1	>6 hours	HPLC
Elia et al 1991	cross sectional	unk	unk	C	healthy	Untreated coeliac disease, patients with acute infections admitted into hospital	Unk	6 hours	2:1	>6 hours	enzymatic analysis
Kuitunen et al 1996	cross sectional	B	0%	B	healthy	Coeliac patients on gluten free diet, coeliac patients given gluten provocation	Gastroenterology department in a university children's hospital	5 hours	2:1	>6 hours	unk
Ukabam et al 1985	cohort	B	unk	B	healthy	Coeliac disease before and after 5-8 months of GFD	Unk	6 hours	2:1	>6 hours	unk
Vilela et al 2008	cross sectional	unk	0%	A	healthy	Coeliac patients after 1 year in GFD, Terminal ileum/ileocolic disease in remission	Gastroenterology clinic in a university hospital	6 hours	2:1	>6 hours	HPLC
Vecsei et al 2009	cohort	B	0%	B	n/a	Newly diagnosed coeliac	Gastroenterology department in a university hospital	5 hours	2:1	unk	HPLC
Johnston et al 2000	cohort	B	unk	B	healthy	Untreated coeliac disease, treated coeliac disease	National screening programme	5 hours	5:2	>6 hours	enzymatic analysis
Hamilton et al 1987	cross sectional	B	unk	C	healthy	Newly diagnosed coeliac disease, subjects with cow milk intolerance, giardiasis	Gastroenterology paediatric clinic in a university hospital	5 hours	5:2	unk	gas-liquid chromatography
Catassi et al 1997	cross sectional	A+B	unk	C	Subjects serologically free of coeliac disease	Subjects with coeliac disease detected by screening	Secondary school screening programme	5 hours	5:2	>6 hours	enzymatic analysis
Van Elburg et al 1993	cross sectional	B	unk	C	healthy	Newly diagnosed coeliac, coeliac in partial remission and not adhering to GFD, relatives of patients with coeliac disease, patients with aspecific GI symptoms	Paediatric department in a university hospital	5 hours	5:2	>6 hours	gas chromatography
Rajani et al 2016	cohort	B+C	unk	A	healthy	Paediatric patients with new serological diagnosis of coeliac disease, paediatric patients with new endoscopic diagnosis of coeliac disease, before and after 1 year of GFD	Coeliac clinic in a children's hospital	unk	5:2	unk	HPLC
Smecuol et al 1999	cross sectional	B	unk	C	n/a	Newly diagnosed coeliac disease patients (mixture of subclinical, asymptomatic, and classical symptoms), first degree relatives of patients with established coeliac disease	Small bowel section of a gastroenterology hospital	unk	5:2	<6 hours	HPLC
Smecuol et al 2005	cross sectional	B+C+D	unk	B	healthy	Patients with coeliac disease on normal diet, dermatitis herpeformis, IgA dermatosis	Small bowel section of a gastroenterology hospital	5 hours	5:2	<6 hours	HPLC
Gatti et al 2013	RCT	B	unk	A	n/a	Coeliac after GFD diet for 2 years with oats, or with placebo (LMR measured before intervention)	Multicentred paediatric gastroenterology services in Italy	5 hours	5:2	>6 hours	HPLC
Smecuol et al 1997	cohort	B+D	unk	D	n/a	Coeliac at baseline and post treatment with 2 months of GFD	Small bowel section of a gastroenterology hospital (both inpatient and outpatient)	unk	5:2	<6 hours	HPLC
Smecuol et al 2013	RCT	C	unk	D	n/a	Active coeliac (mixture of subclinical and symptomatic coeliac disease) given probiotic or treated with placebo (LMR measured before intervention)	Small bowel section of a gastroenterology hospital	5 hours	5:2	>6 hours	HPLC

Juby et al 1989	case control	B+D	unk	C	healthy	Newly diagnosed coeliac disease before given GFD	General gastroenterology clinic in a university hospital	5 hours	5:2	>6 hours	HPLC

A) European Society of Paediatric Gastroenterology criteria
B) Small bowel biopsies
C) Serological diagnosis
D) Clinical features

A) >6 months GFD
B) Unknown/varying duration of GFD
C) Untreated coeliac disease
D) <6 months GFD

Table S2. Summary of studies of gut permeability in coeliac disease. Methods of diagnosis were separated into five categories (A-E) as defined at the base of the column. Management was separated into four categories (A-D) as defined in the key at the base of the column. Abbreviations: RCT – Randomised Control Trial, CDAI – Crohn's Disease Activity Index, unk – unknown, n/a – not applicable, GFD – Gluten Free Diet, HPLC – High Performance Liquid Chromatography, NBM – nil by mouth.

**Data**	**Variation**
Study design	Cross sectional, cohort, case control, randomised control trials
Sample size	Is the sample size appropriate, and has a power calculation been performed?
Participant selection	Is the inclusion and exclusion criteria the same in all the studies? In some studies, some patients had history of surgery. Patients were diagnosed either through CDAI criteria, PCDAI criteria, clinically, endoscopically, radiologically, via histology, or the modified Harvey Bradshaw index
Control selection	Was there any variation in selection of control population in all studies, and were they all healthy controls?
**Methods**	
Populations and patient characteristics	Patients were either in remission, active or relapsing disease. Crohn's patients were managed by either aminosalicylates, azathiopine, steroids, thalidomide, metronidazole, not on medications, sulphursalazine, hematinics/multivitamins/calcium supplements or any combination of above. Coeliac patients had different lengths of gluten free diet regime.<br>Have premucosal factors (incomplete ingestion of test solution, vomiting, gastric emptying, dilution by secretion, rate of transit) or disposal factors (systemic distribution, solute metabolism, rate of renal clearance) been addressed?
Disease relapse	Any mention of patients who relapsed, or definition of relapse explicitly mentioned in study?
Type of compounds	What is the ratio of lactulose: mannitol given in the solute? Some studies used a L:M ratio of 5:2, or 2:1 or a combination. Some solutes have additional compounds such as sucrose. Is the solute osmolality different between studies?
Study protocol when drinking solute	There is a variation of NBM period (<6 hours or > 6 hours)
Study protocol after drinking solute	There is a variation in urine collection period- 5, 6, 7 or 12 hours
Urine storage	Is there uniformity in how the urine was stored, and the temperature used to keep the urine before analysis?
Urine analysis	Urine analysed either using HPLC, gas/liquid chromatography, or enzymatic analysis
Statistical analysis	Different forms of statistical analysis used in interpreting the results
**Results**	
How results were presented	Was the mean or median used, and was the standard deviation or range also used as well?
ROC curve	Only 1 out of 9 studies used the ROC curve to measure sensitivity/specificity

Table S3. Sources of variability for the studies included in the meta-analysis.

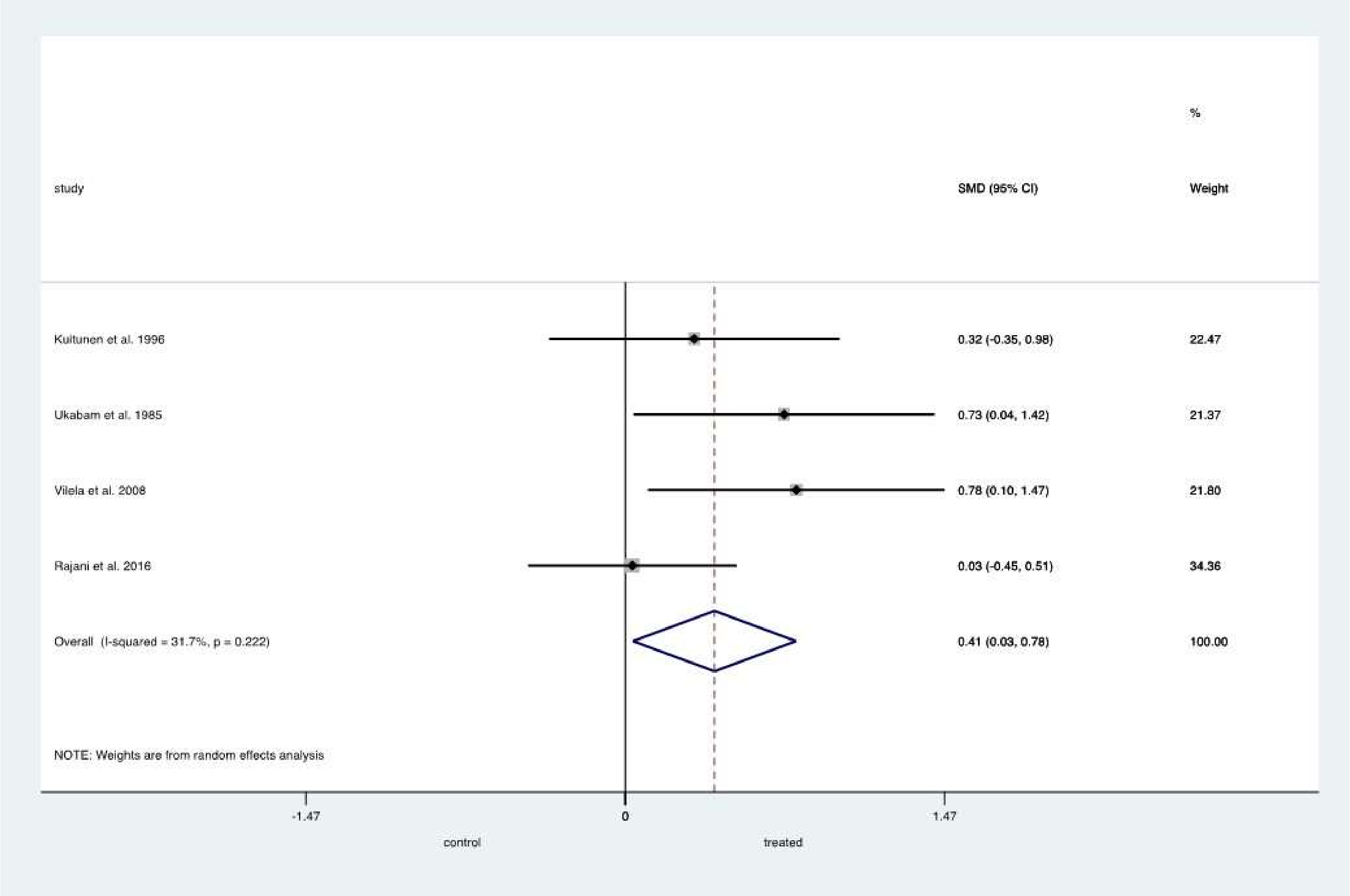

Fig. S3(A). Standard Mean Difference (SMD) in LMR between treated coeliac disease and healthy controls.

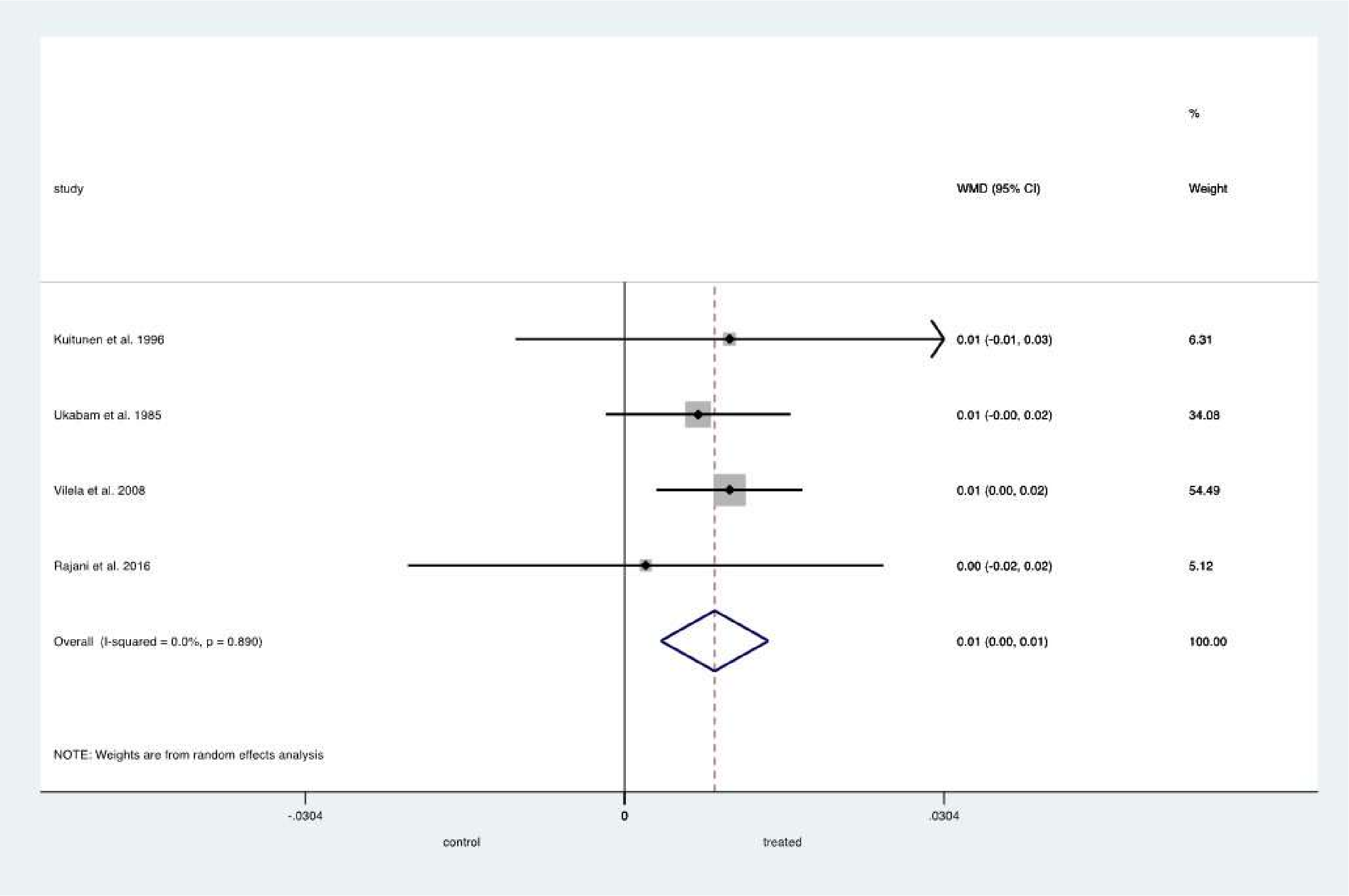

Fig. S3(B). Weighted Mean Difference (WMD) in LMR between treated coeliac disease and healthy controls.

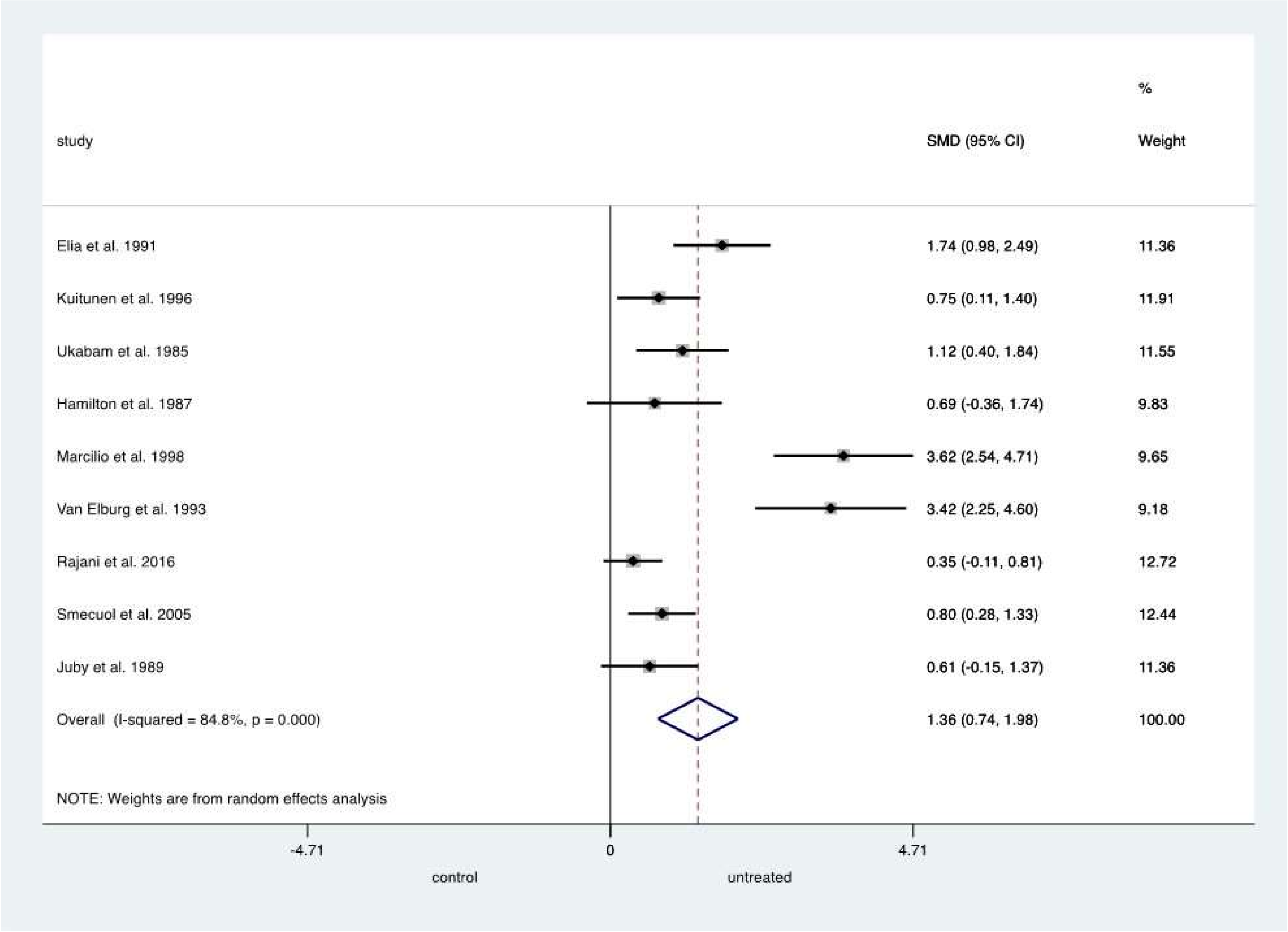

Fig. S3(C). Standard Mean Difference (SMD) in LMR between untreated coeliac disease and healthy controls.

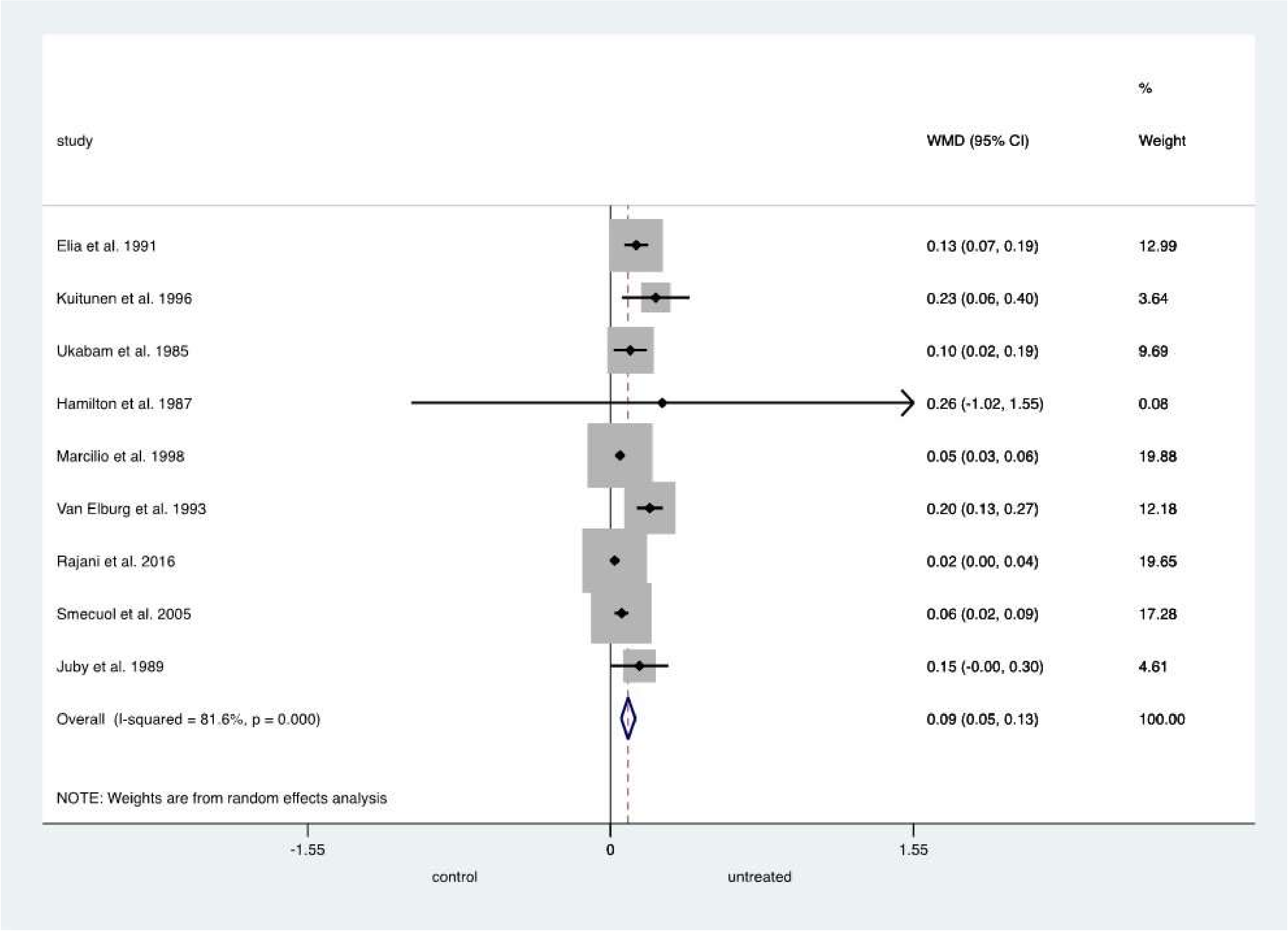

Fig. S3(D). Weighted Mean Difference (WMD) in LMR between untreated coeliac disease and healthy controls.

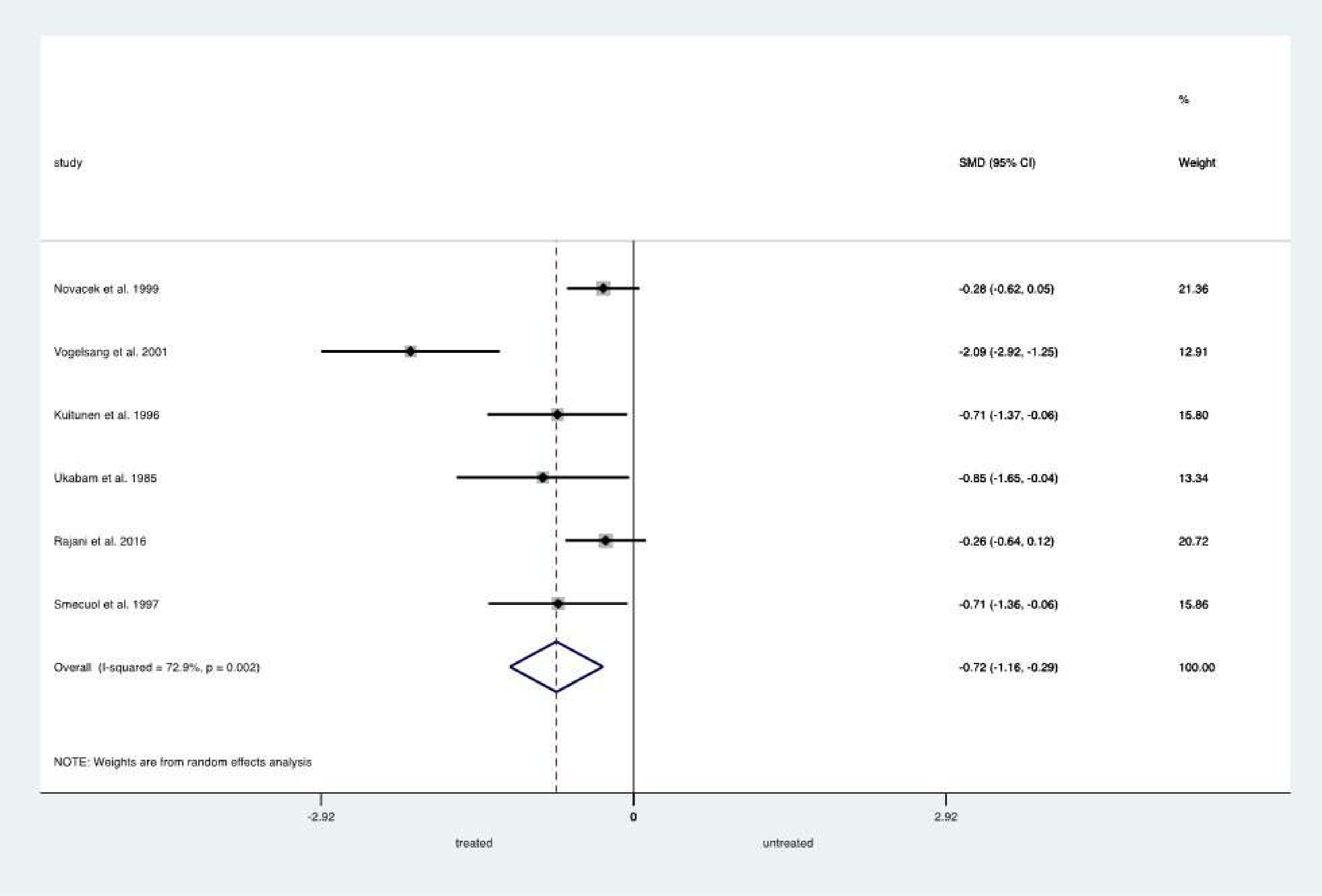

Fig. S3(E). Standard Mean Difference (SMD) in LMR between untreated and treated coeliac disease.

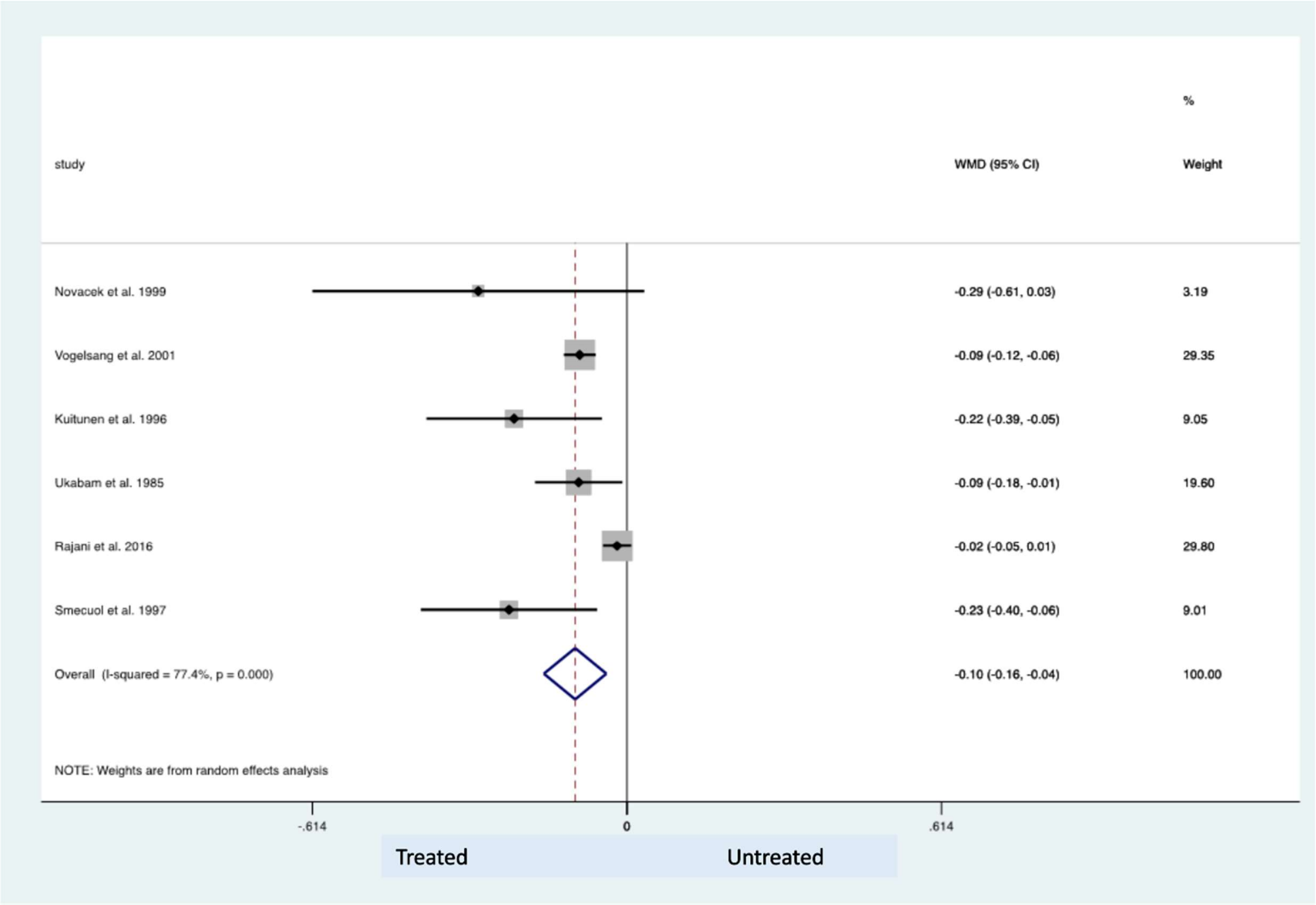

Fig. S3(F). Weighted Mean Difference (WMD) in LMR between untreated and treated coeliac disease.

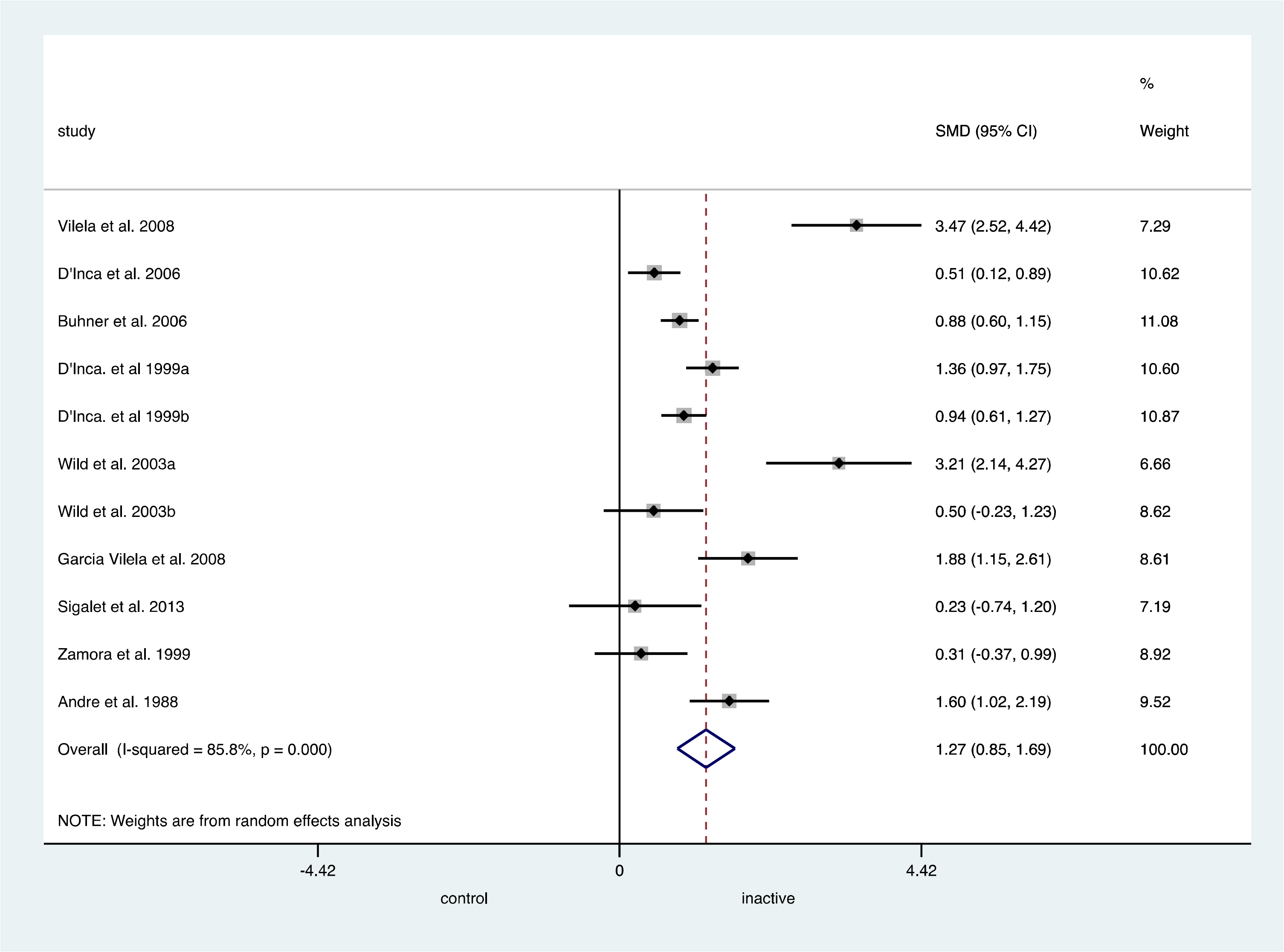

Fig. S4(A). Standard Mean Difference (SMD) in LMR between healthy controls and inactive Crohn's disease.

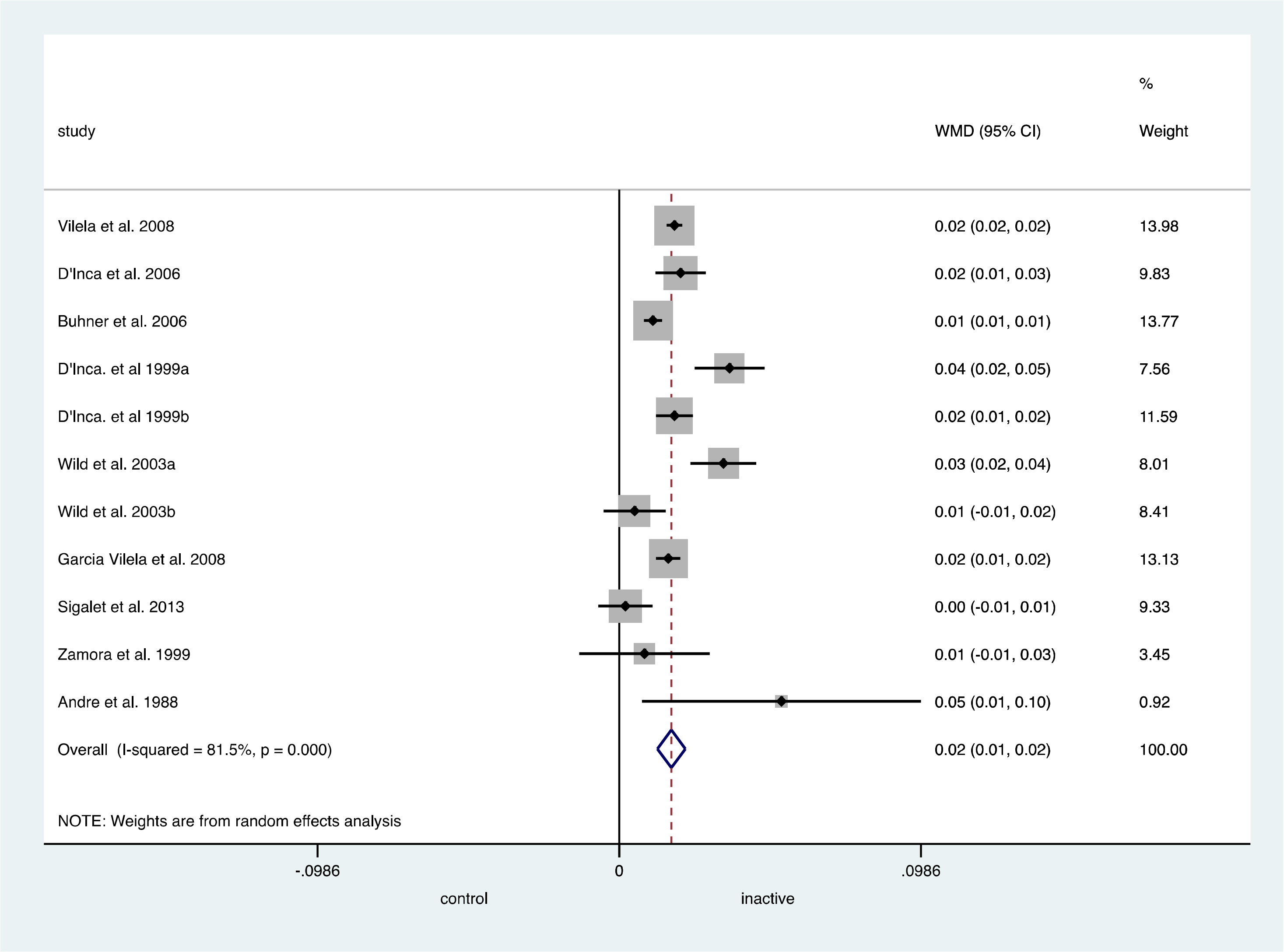

Fig. S4(B). Weighted Mean Difference (WMD) in LMR between healthy controls and inactive Crohn's disease.

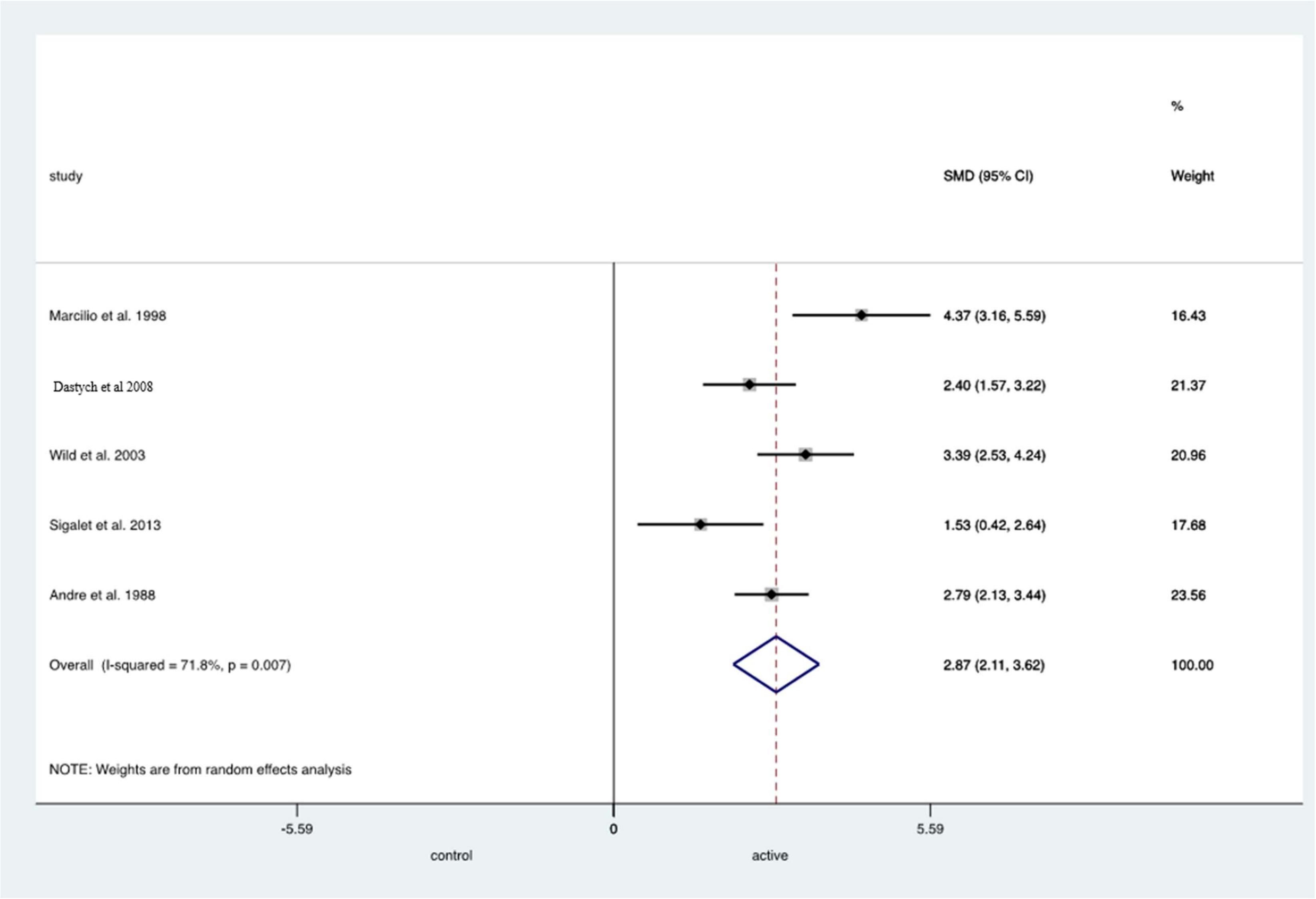

Fig. S4(C). Standard Mean Difference (SMD) in LMR between active Crohn's disease and healthy controls.

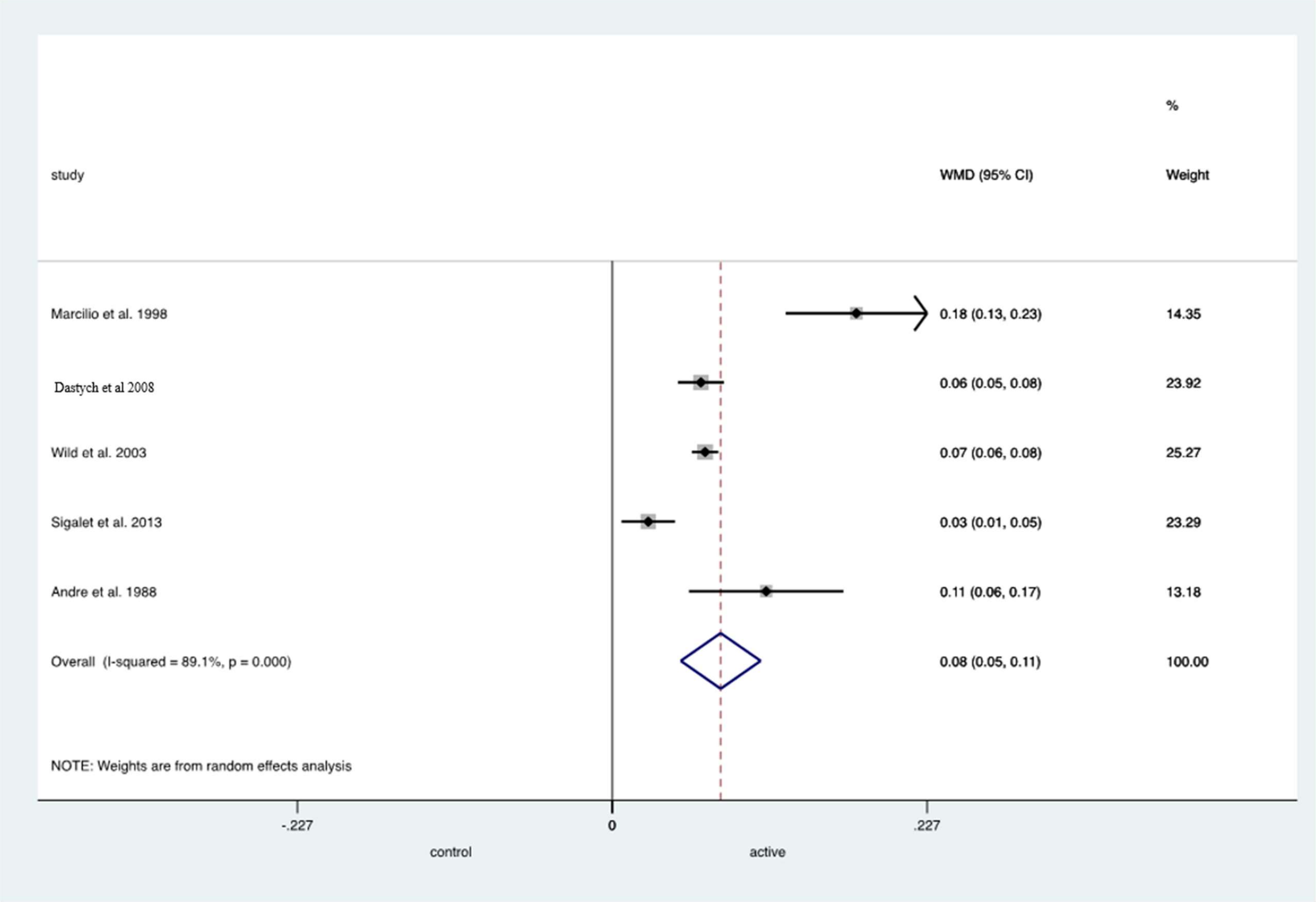

Fig. S4(D). Weighted Mean Difference (WMD) in LMR between active Crohn's disease and healthy controls.

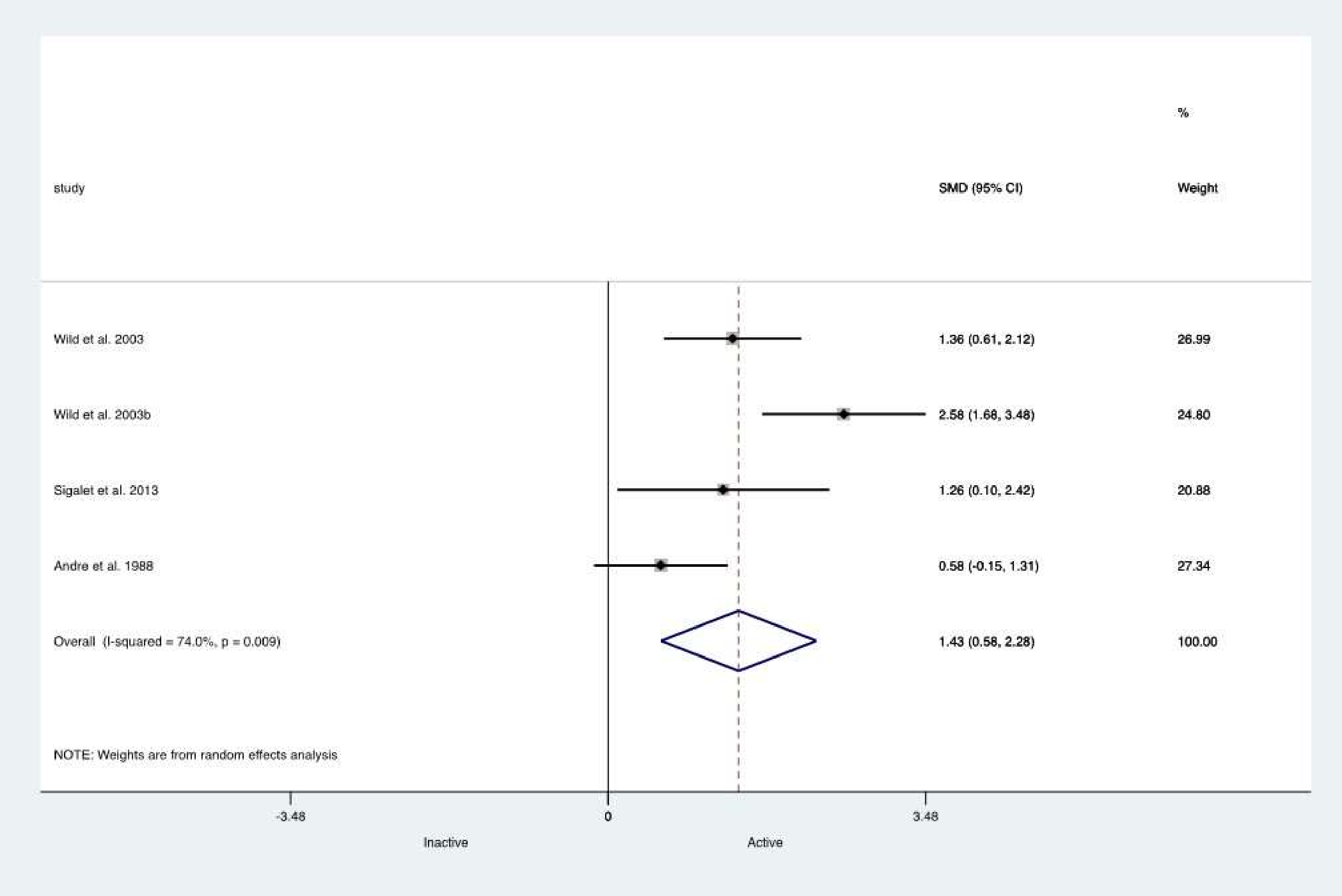

Fig. S4(E). Standard Mean Difference (SMD) in LMR between active and inactive Crohn's disease.

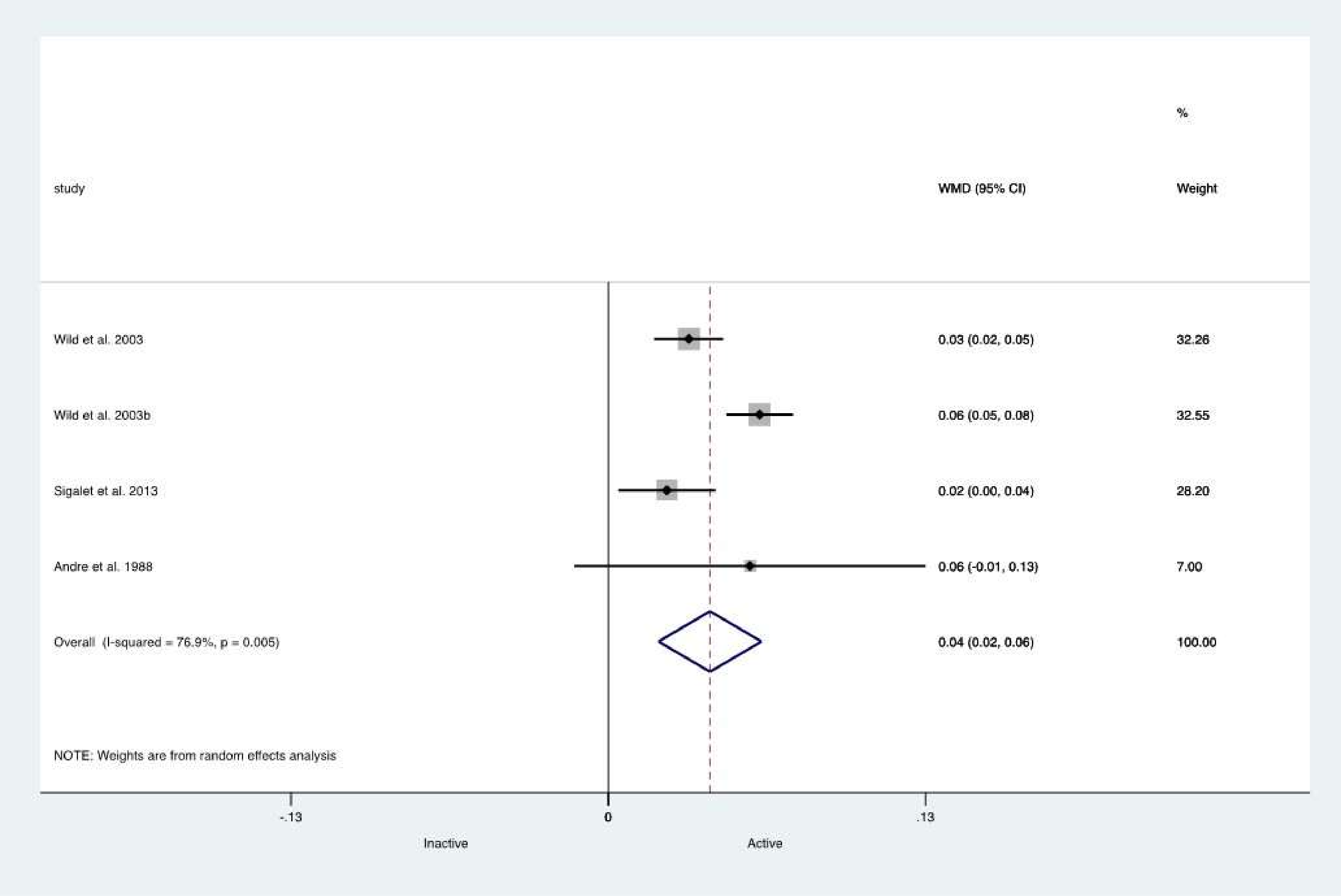

Fig. S4(F). Weighted Mean Difference (WMD) in LMR between active and inactive Crohn's disease.

Study	Total Number of Patients	Cut-off value for coeliac disease diagnosis	Sensitivity (%)	Specificity (%)	Clinical Relevance	How cut-off is derived
Juby et al.1989	44	0.028	89	54	Screening of coeliac disease in general population	Mean + 2 SD of a previous Cellubiose/Mannitol (Ce/Ma) study
Johnston et al.2000	77	0.024	87	71	Screening of coeliac disease in general population	Mean + 1.28 SD of log transformed LMR (representing the 90th percentile) for healthy volunteer group
Catassi et al.1997	29	0.044	45	Not Reported	Screening of coeliac disease in general population	Mean + 1.65 SD of the square root of LMR
Smecuol et al.1999	41	0.025	100	83	Screening of coeliac disease among first degree relatives	Not Reported

Table S4. Studies depicting sensitivity and specificity of LMR in screening for coeliac disease. Abbreviations: SD – standard deviation. LMR: Lactulose Mannitol Ratio

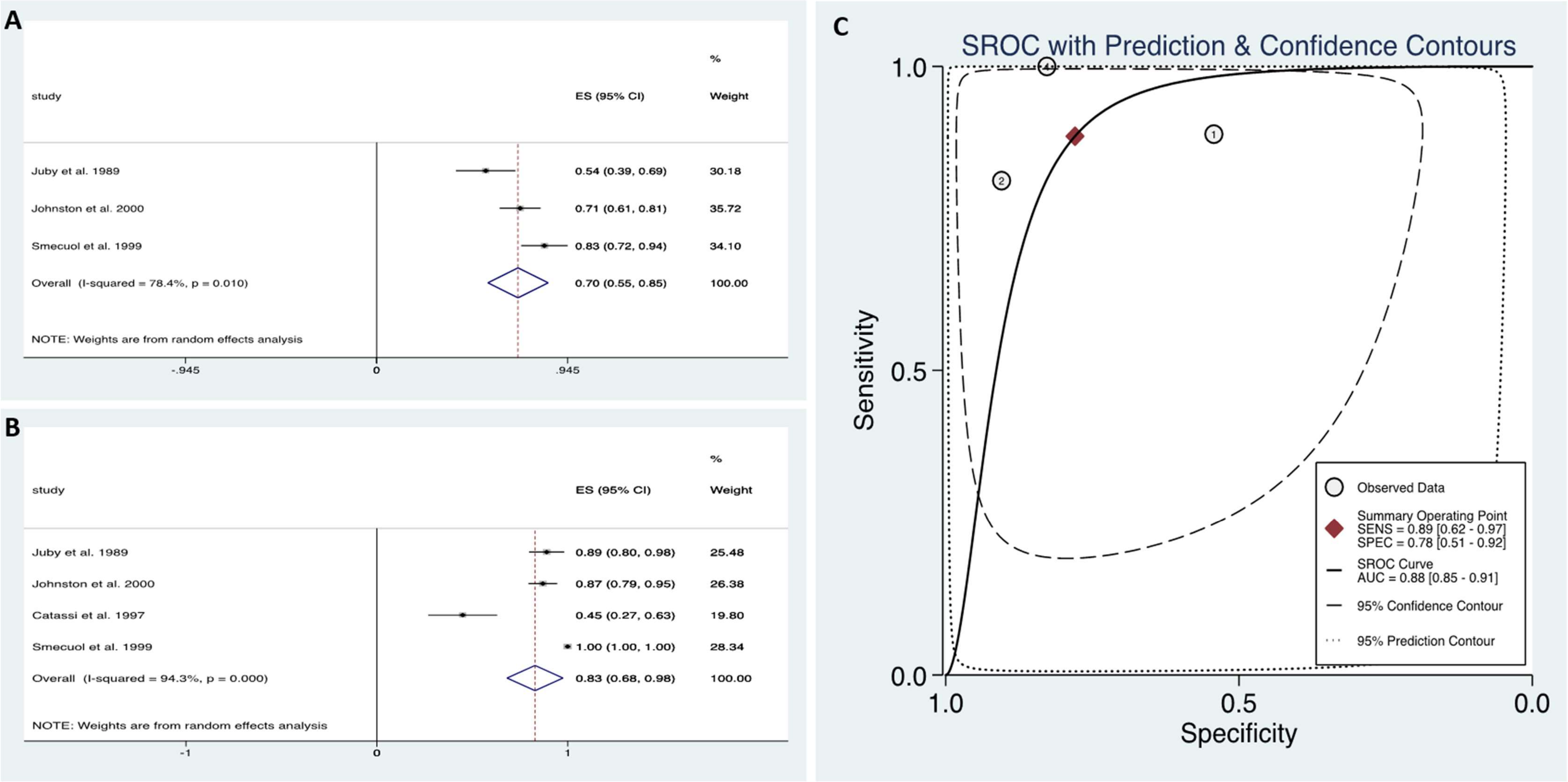

Fig. S5. Sensitivity and specificity of the L:M test in coeliac disease. (A) Pooled specificity values for the L:M test in coeliac disease. (B) Pooled sensitivity values for the L:M test in coeliac disease. (C) Summary Receiver Operating Characteristic (SROC) curve for coeliac disease, showing prediction and confidence contours.

Table B1. Newcastle Ottawa Score Assessing Risk of Bias for Case Control Studies (denotes 1 point).*

	Selection				Comparability	Exposure			
**Study**	Is the case definition adequate?	Representativeness of the cases	Selection of controls	Definition of controls	Comparability of cases and controls on basis of the design or analysis	Ascertainment of exposure	Same method of ascertainment for cases and control	Non-response rate	**Total score**
Zamora et al 1999		*	*	*	**	*	*	*	8
Hilsden et al 1996			*	*		*	*		4
Swanson et al 2011	*			*	*	*	*	*	6
Juby et al 1989	*	*	*	*		*	*	*	7
Breslin et al 2001	*		*	*	**	*	*	*	8

Table B2. Newcastle Ottawa Score Assessing Risk of Bias for Cross Sectional Studies (denotes 1 point).*

	Selection				Comparability	Outcome		
**Study**	Representativeness of the sample	Sample size	Ascertainment of exposure	Presence of Non-respondents	The subjects in different outcome groups are comparable based on the study design or analysis, Confounding factors are controlled	Assessment of outcome	Presence of Statistical test	**Total score**
Catassi et al 1997	*	*	**		*	**		7
Elia et al 1991			*		*	**		4
Hamilton et al 1987			**			**		4
Marsilio et al 1998			**			**		4
Vogelsang et al 2001	*		**			**	*	6
Van Elburg et al 1993	*		**			**		5
Kuitunen et al 1996	*		**			**	*	6
Andre et al 1988		*	**			**		5
Vilela et al 2008			**			**	*	5

Dastych et al 2008		*	*			**	*	5
D'Inca et al 2006	*	*	**			**	*	7
Buhner et al 2006	*	*	**			**	*	7
Smecuol et al 2005	*	*	*		*	**	*	7
Smecuol et al 1999	*		**			**	*	6

Table B3. Risk of Bias for Randomised Control Trials (RCT) using the Cochrane Risk of Bias Tool

Study	Random sequence generation (selection bias)	Allocation concealment	Blinding of participants and personnel	Blinding of outcome assessment (detection bias)	Incomplete outcome data (attrition bias)	Selective reporting (reporting bias)	Overall
Smecuol et al 2013	No information	Low	Low	No information	Low	Low	Low
Garcia Vilela et al 2008	Low	High	High	High	Low	Low	High
Benjamin et al 2012	Low	Low	High	Low	Low	Low	Moderate
Gatti et al 2013	No information	No information	Low	No information	High	Low	Moderate

Table B4. Risk of bias in non-randomised trials and cohort studies using the ROBINS-I score.

	**Pre-intervention**		**At intervention**	**Post intervention**				
**Study**	Bias due to confounding	Bias in selection of participants into the study	Bias in classifications of interventions	Bias due to deviations from intended interventions	Bias due to missing data	Bias in measurement of outcome	Bias in selection of the reported result	**Overall bias**
Zamora et al 1999	Low	Low	Low	Low	Low	Moderate	Moderate	Moderate
Sturniolo et al 2001	Low	Moderate	Low	Moderate	Low	Serious	Moderate	Serious

Hilsden et al 1996	Low	Moderate	Low	Moderate	Moderate	Serious	Moderate	Serious
Swanson et al 2011	Low	Moderate	Low	Low	Low	Moderate	Low	Moderate
Wild et al 2003	Low	Serious	Low	Low	Low	Serious	Moderate	Serious
Ukabam et al 1985	Moderate	Serious	Low	Low	Low	Low	Moderate	Moderate
Johnston et al 2000	Low	Moderate	Low	No information	Low	Low	Moderate	Moderate
Novacek et al 1999	Moderate	Serious	Low	Low	Moderate	Low	Low	Moderate
Rajani et al 2016	Low	Moderate	Low	Low	Moderate	Low	Low	Moderate
Vecsei et al 2008	Low	Moderate	Low	Low	Moderate	Low	Moderate	Moderate
Sigalet et al 2013	Low	Moderate	Low	Low	Low	Low	Moderate	Low
D'Inca et al 1999	Moderate	Moderate	Moderate	No information	Low	Low	Low	Moderate
Smecuol et al 1997	Low	Moderate	Low	Low	Moderate	Low	Serious	Serious
Hilsden et al 1999	Low	Moderate	Moderate	Moderate	Low	Low	Low	Moderate

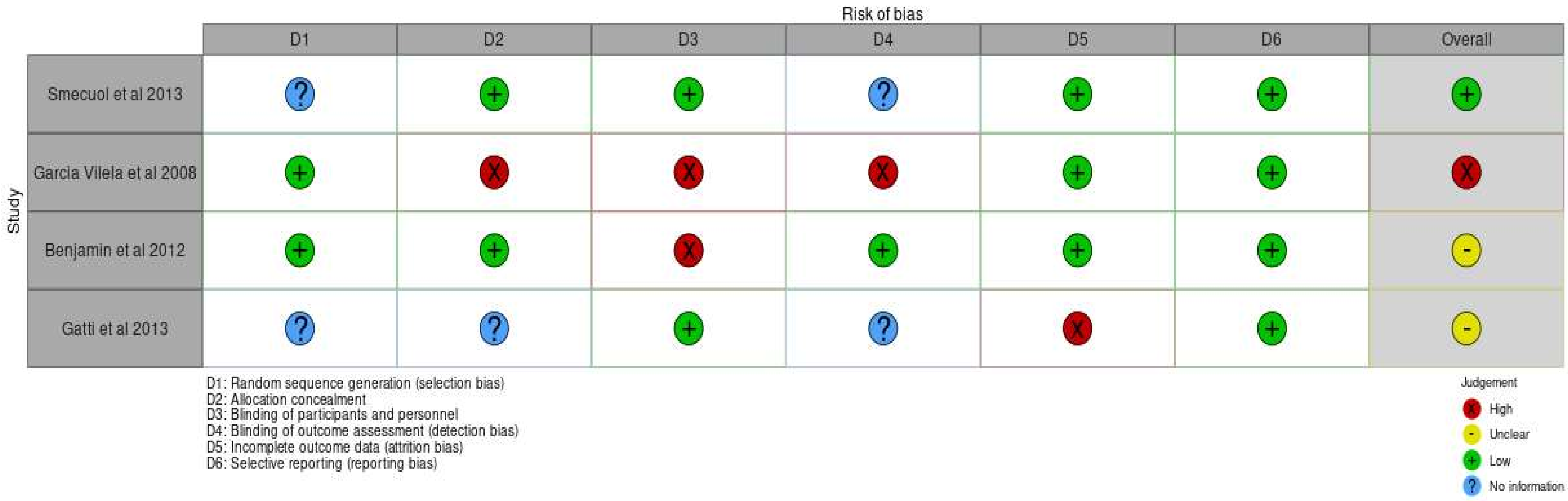

Figure S6: Risk of bias for each risk of bias item in RCT studies

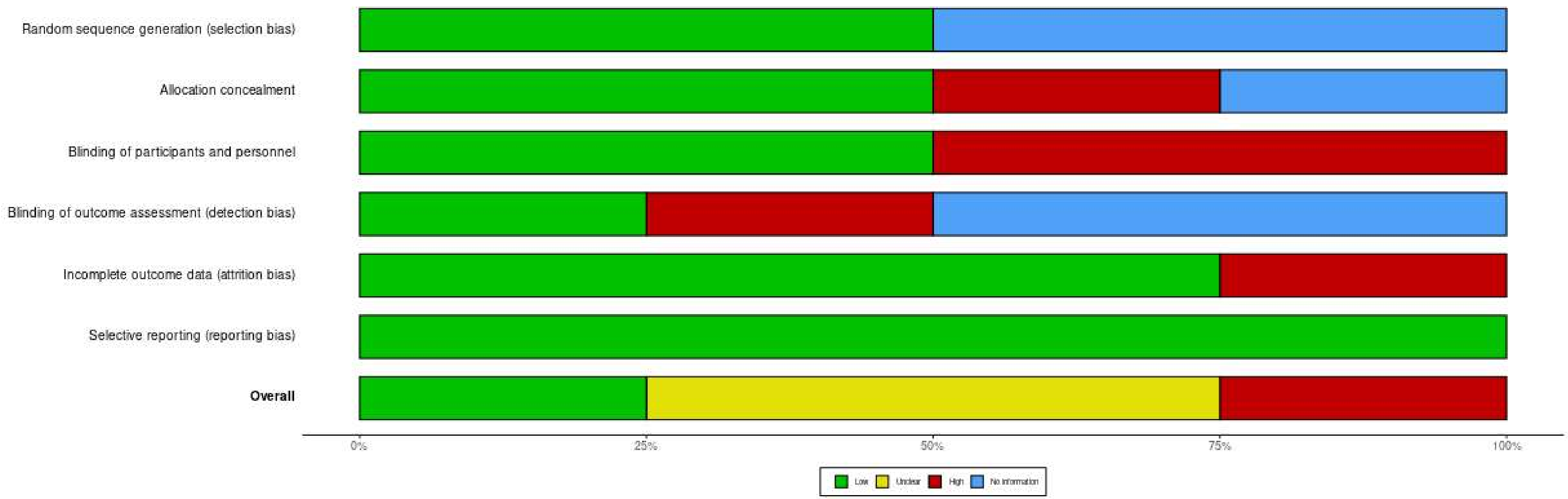

Figure S7: Risk of bias assessments presented per risk of bias domain in RCT studies

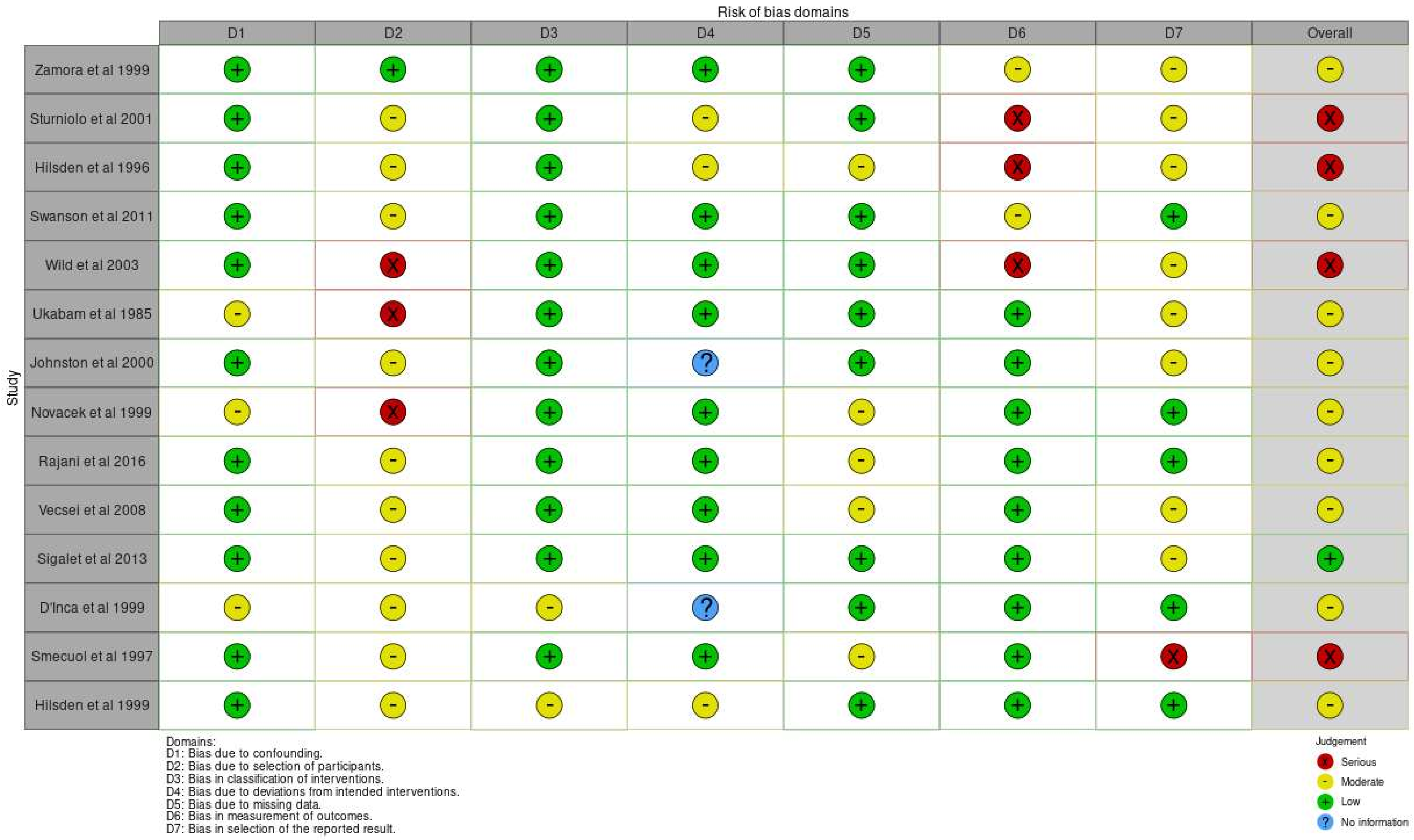

Figure S8: Risk of bias for each risk of bias item in non-randomised and cohort studies

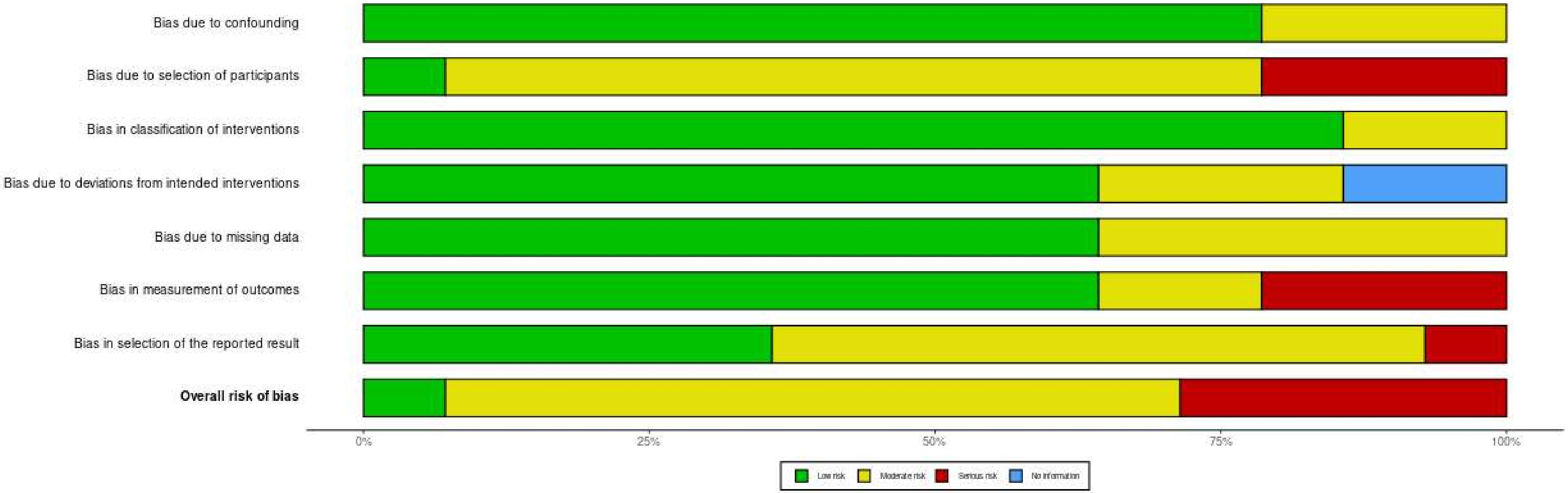

Figure S9: Risk of bias assessments presented per risk of bias domain in non-randomised and cohort studies
